# Geographic Information System Applications in Bee Research

**DOI:** 10.3390/insects17060566

**Published:** 2026-05-29

**Authors:** Nilton B. Rojas-Briceño, Jhonsy O. Silva-López, Betty K. Guzman, Manuel A. Ix-Balam, José L. Ramos-Tejeda, Manuel Oliva-Cruz, Jaris Veneros, Ligia García

**Affiliations:** 1 Programa de Doctorado en Ciencias para el Desarrollo Sustentable, Escuela de Posgrado, Universidad Nacional Toribio Rodríguez de Mendoza de Amazonas, Chachapoyas 01001, Peru; 2 Grupo de Investigación en Ciencia de la Información Geoespacial, Escuela Profesional de Ingeniería Ambiental, Universidad Nacional de Moquegua, Pacocha 18610, Peru; jramost@unam.edu.pe (J.L.R.-T.); 3 Instituto de Investigación para el Desarrollo Sustentable de Ceja de Selva, Universidad Nacional Toribio Rodríguez de Mendoza de Amazonas, Chachapoyas 01001, Peru; jhonsy.silva@untrm.edu.pe (J.O.S.-L.); manuel.ix@untrm.edu.pe (M.A.I.-B.); manuel.oliva@untrm.edu.pe (M.O.-C.); ligia.garcia@untrm.edu.pe (L.G.); 4 Área de Cartografía y Teledetección, Laboratorio de Agrostología, Instituto de Investigación en Ganadería y Biotecnología, Facultad de Ingeniería Zootecnista, Agronegocios y Biotecnología, Universidad Nacional Toribio Rodríguez de Mendoza de Amazonas, Chachapoyas 01001, Peru; 5 Estación Experimental Agraria Moquegua, Dirección de Investigación y Desarrollo Tecnológico, Instituto Nacional de Innovación Agraria (INIA), Panamericana Sur Km 4.5, Moquegua 18001, Peru; bguzman@inia.gob.pe (B.K.G.)

**Keywords:** apiculture, beekeeping, bees, floral resource, Geographic Information Systems, honey bee, multicriteria decision analysis, pollinators, remote sensing, spatial analysis

## Abstract

Bees play crucial ecological, economic, and environmental roles, and research on them increasingly includes a spatial dimension. Geographic Information Systems (GISs) enable the acquisition, storage, analysis, management, and visualization of spatial data. However, GIS applications in bee research have expanded while remaining dispersed across topics, tools, taxa, and methodological approaches. This study provides a comprehensive and updated review of GIS applications in bee research by integrating bibliometric analysis with a structured synthesis of GIS purposes and techniques. A total of 228 publications were analyzed to assess publication trends, co-authorship patterns, keyword themes, study areas, taxonomic coverage, GIS application themes, and methodological tools. GIS was used to select suitable apiary sites, map floral resources, analyze bee behavior, assess diseases and pests, monitor bee products, evaluate urban and landscape contexts, and predict climate change effects. The main GIS-related approaches included multicriteria decision analysis (MCDA), remote sensing, species distribution models (SDMs), spatial interpolation, WebGIS platforms, and emerging machine-learning applications. The review also identified underrepresented taxa, especially wild bees, stingless bees, and other *Apis* species. Future advances should integrate MCDA with data-driven models, improve floral-resource mapping with remote sensing, and strengthen reproducibility through standardized spatial data and workflows.

## Simple Summary

Bees are essential for pollination, food production, biodiversity, and beekeeping. Because bees, flowers, apiaries, pests, diseases, and environmental risks all have a spatial component, Geographic Information Systems (GISs) are increasingly used to study and manage bee-related problems. This review analyzed 228 publications that applied GIS or related geospatial methods in bee research. The studies showed that GIS has been used for many purposes, including selecting suitable apiary locations, mapping floral resources, analyzing bee diseases and pests, studying bee movement, estimating pollination services, monitoring bee products, and predicting the effects of climate change. Most studies focused on beekeeping systems and *Apis mellifera*, while wild bees, stingless bees, and other bee groups were less represented. The review also showed that GIS applications are expanding with new tools such as remote sensing, species distribution models, WebGIS, machine learning, and decision-support systems. Overall, GIS provides useful tools for researchers, beekeepers, and decision-makers, but future studies should improve data quality, reproducibility, and taxonomic coverage.

## 1. Introduction

Bees play crucial ecological and economic roles as essential pollinators of wild plants and agricultural crops [1,2]. They are also central to beekeeping, an important agricultural activity that contributes to rural economic development [3,4] through pollination services [5] and the production of goods relevant to food and human health [6]. In addition, bees collect and accumulate materials and particles from the environment, making them valuable bioindicators for monitoring large areas, including agricultural zones [7] and urban areas [8]. However, bees face critical conservation threats due to climate change, anthropogenic land-use change, habitat loss, and other factors [9,10,11]. As a result, research on bees has increased substantially worldwide [12].

Ecological studies often include a spatial dimension, such as species geolocation, migratory routes, habitat distribution, and the monitoring of climate change impacts [13]. Consequently, Geographic Information Systems (GISs) have become essential in ecological studies, as they allow the exploration of spatial relationships within and between datasets. These spatial relationships enable the quantification, interpretation, and explanation of biogeographic and ecological patterns [14]. As systems comprising hardware and software components, GIS facilitate the acquisition, storage, manipulation, analysis, management, and presentation of spatial or geographic data. GIS has been widely applied across various disciplines to analyze spatial patterns and distributions and to understand interactions between geographic and non-geographic entities in space and time [15].

In bee research, GIS has been applied to diverse purposes, including selecting suitable apiary locations [16], mapping plants of interest to bees [17], studying bee behavior [18], analyzing honey bee diseases and pests [19], and predicting the effects of climate change on bees [10]. Although a previous narrative review noted that GIS had been used in relatively few studies related to honey bees and beekeeping [20], the current literature shows a growing but dispersed body of applications across topics, tools, taxa, and methodological approaches. Therefore, an updated and structured synthesis of GIS applications in bee research is warranted.

Previous reviews have addressed related aspects of GIS use in apiculture and bee research. Rogers et al. [21,22] provided a methodological and tutorial-oriented contribution introducing basic GIS principles, data types, and spatial analysis procedures for honey bee research. Abou-Shaara [20] conducted a narrative review exploring GIS applications in honey bees, beekeeping, and bee products. Kotovs and Zacepins [23] reviewed seven selected studies on GIS applications in beekeeping, focusing on the practical problems these tools were used to address. In addition, Alleri et al. [24] conducted a systematic review on precision beekeeping, including a brief dedicated subsection on GIS applications in apiculture. However, these studies do not provide an integrated and updated assessment that combines bibliometric patterns with a structured synthesis of the main purposes and tools of GIS use in bee research. In this context, the present study addresses this gap by examining publication trends and collaboration patterns, while systematically summarizing GIS applications for bees across diverse purposes and tools.

The objectives of this study are twofold: (i) to carry out a bibliometric analysis to explore co-authorship among countries, authors, and institutions, and to identify trends in publications and keyword themes; and (ii) to examine and summarize the purposes and GIS tools used in the published literature on bees, providing insights to guide future studies and highlight emerging initiatives.

## 2. Materials and Methods

### 2.1. Publication Search and Selection

**Figure 1:**
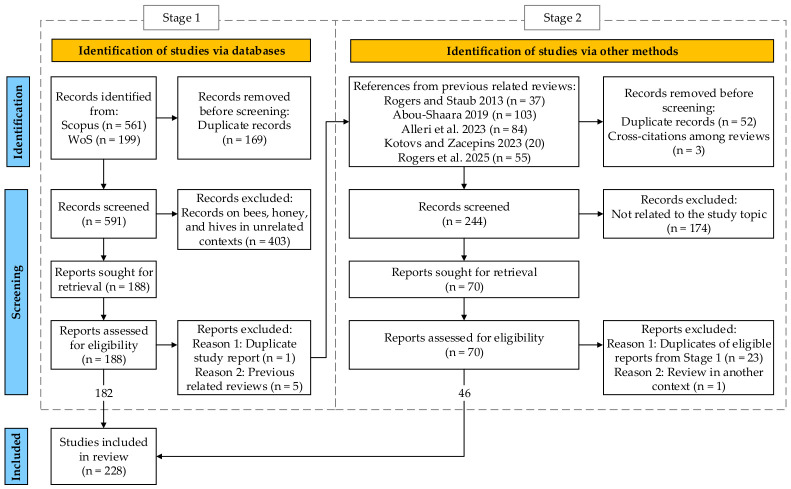
Flow of steps for the systematic literature review. The previous related reviews used in Stage 2 were Abou-Shaara [20], Rogers and Staub [21], Rogers et al. [22], Kotovs and Zacepins [23], and Alleri et al. [24].

A systematic literature search approach was used, following the identification, screening, and eligibility procedures described in the Preferred Reporting Items for Systematic Reviews and Meta-Analyses (PRISMA) [25]. In stage 1 (Figure 1), identification was performed on 27 April 2026, in the Scopus and Web of Science databases, considering titles, abstracts, and keywords. These databases were chosen because they include a wide variety of scientific literature in different fields of knowledge and their indexed publications undergo a formal and rigorous peer-review process [26,27]. A search was performed using Boolean operators (AND and OR), bee-related words, and GIS-related terms. The syntax was (“*bee” OR “honey*” OR “hive” OR “apicultur*” OR “beekeep*” OR “apiari*” OR “apiary*”) AND (“gis” OR “geograph* information system*” OR “geomatic*” OR “geoinformat*” OR “geospat*”), with truncations to expand the results. The results of each database were exported in CSV format, combined, and duplicate documents were eliminated (n = 169). The remaining 591 documents were examined by reading their titles and abstracts for screening. This screening was performed by two members of the research team. When disagreements arose regarding whether to include or exclude a document, a third member was consulted to reach the final decision. A total of 403 studies were excluded, mainly because the terms “bee”, “honey”, or “hive” were used in contexts outside the objectives of this study, such as Zigbee, artificial bee colony algorithms, and honeycomb-like engineering structures.

The 188 previously selected articles were assessed for eligibility by reading the full texts. Inclusion criteria were: (i) the publication was an original study and not a review; (ii) the full text of the article was available; and (iii) the article deals with a spatial analysis related to bees, either directly or indirectly through spatial-context variables used to interpret bee- and apiary-related patterns, and shows a map as a result or explicitly reports the use of GIS, spatial modeling, or spatial processing in the methodological approach. Six reports were excluded at this stage, resulting in 182 selected studies. The excluded studies include five previous reviews related to the objective of this study [20,21,22,23,24]. Therefore, the identification, screening, and eligibility procedures were applied to the bibliographic references of these previous reviews. In stage 2 (Figure 1), 299 references were examined; after removing duplicates and cross-citations, 244 records were screened using the same criteria as in stage 1. Of these, 70 reports were assessed for eligibility, and 46 additional studies were selected. Thus, 228 publications were included for analysis in this study, and the complete list is provided as Supplementary Materials.

### 2.2. Bibliometric Analysis

Publication growth was described using annual publication counts and the compound annual growth rate (CAGR), calculated as [(*N_final_*/*N_initial_*)*^1/t^* − 1] × 100, where *N_initial_* is the number of publications in the initial year, *N_final_* is the number of publications in the final year, and *t* is the number of years between both observations [27]. CAGR was calculated for 2000–2025 to summarize the main growth period, excluding the incomplete 2026 record. Bibliometric analyses were performed using VOSviewer v1.6.20 [28] and the Bibliometrix package v5.4.0 [29].

VOSviewer was used to generate co-authorship networks for countries (≥2 publications), institutions (≥3 publications), and authors (≥2 publications), as well as co-occurrence maps of author keywords (≥3 occurrences). All VOSviewer analyses were performed using the full counting method. Network maps and clusters were generated using VOSviewer’s default normalization and clustering procedures. Before network construction, separate thesaurus files were prepared for keywords, institutions, and authors [30]. For keyword harmonization, hyphens and slashes were replaced with spaces, plural forms were singularized, and equivalent terms were merged; for example, “geographic information system”, “geographical information systems”, and “gis” were standardized as “GIS”. For authors, alternative spellings, initials, and name variants were merged under a single standardized form. For institutions, affiliation variants, institutional subunits, and alternative names were standardized. This procedure reduced artificial inflation of counts and improved the consistency of rankings and co-occurrence networks.

The Bibliometrix package, through the Biblioshiny interface, was used to evaluate author productivity, identify core sources, and construct the thematic map. Author productivity was assessed using Lotka’s Law [31] by comparing the empirical distribution with both the theoretical inverse-square model (β = 2) and the fitted model, using Kolmogorov–Smirnov goodness-of-fit tests. Core sources were identified using Bradford’s Law [32] by ranking sources by decreasing productivity, fitting the Bradford distribution to the cumulative number of articles, and dividing sources into Bradford zones with approximately one-third of the articles per zone. The thematic map was constructed from author keywords using the same harmonized keyword set applied in the VOSviewer analysis. For this analysis, 600 terms were considered, with a minimum cluster frequency of 5 per thousand documents, an alpha parameter of 0.5, and the Louvain clustering algorithm. Callon’s centrality and density were used to characterize the strategic position of thematic clusters [33]. Centrality was interpreted as an indicator of the importance of a topic within the overall field, whereas density was interpreted as an indicator of the internal development of the topic [34].

### 2.3. Thematic Analysis

The 228 selected studies were classified through a structured full-text review according to the main topic addressed, understood as the primary purpose of GIS use, and the main GIS tool or methodological approach applied. The methodological details of the main topics and tools were then summarized, including a methodological flow summary. This is intended to guide future studies and to highlight recent and novel initiatives. In addition, the country of study and the geographic scale of analysis were recorded to characterize the spatial scope of the reviewed literature. The bee taxa or taxonomic groups addressed in each study were also extracted to incorporate the biological scope of GIS applications in bee research.

## 3. Results

### 3.1. General Characteristics of the Reviewed Studies

#### 3.1.1. Publication Trends and Study Scope

**Figure 2:**
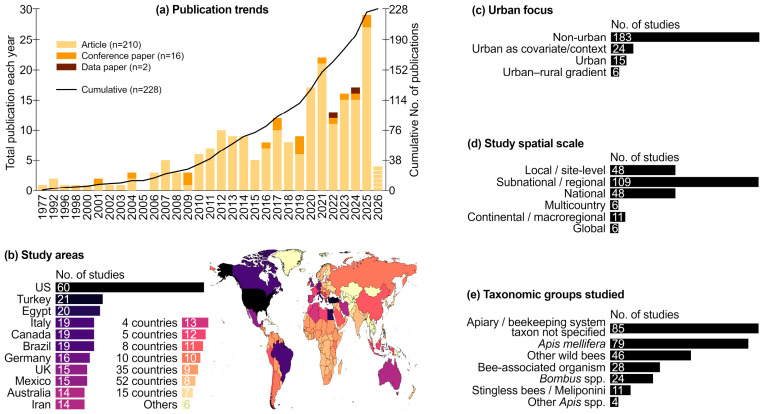
Publication trends and scope of GIS applications in bee research, including annual publication trends and cumulative publications (**a**), countries used as study areas (**b**), urban focus (**c**), spatial scale (**d**), and taxonomic groups studied (**e**).

The annual trend of publications showed that 2025 (n = 29) and 2021 (n = 22) recorded the highest productivity, while the first publication dated to 1977 [35] (Figure 2a). From 2000 to 2025, the field showed a CAGR of 14.42%, indicating a marked expansion of GIS-related applications in bee research. Regarding study areas, the United States (US) was the country where the largest number of studies was conducted (n = 60), followed by Turkey (n = 21), Egypt (n = 20), and Italy, Canada, and Brazil (n = 19 each) (Figure 2b). Most studies were classified as non-urban (n = 183), although urban contexts were also analyzed as the main study focus, for example, in studies on urban bee communities, green roofs, and urban beekeeping [36,37,38,39], as urban–rural gradients [40,41,42], or as explanatory covariates in landscape analyses [43] (Figure 2c). GIS allowed studies at different geographic scales; most studies were conducted at subnational/regional scales (n = 109) [44,45], followed by national (n = 48) [2,46,47] and local/site-level studies (n = 48), including areas surrounding one or more apiaries [48,49,50]. Global [51,52], multicountry [53,54], and continental/macroregional studies [55,56] were less frequent (Figure 2d).

#### 3.1.2. Taxonomic Groups Studied

The reviewed studies were dominated by two categories: beekeeping systems without explicit taxonomic specification (n = 85) and studies explicitly focused on *Apis mellifera* (n = 79) (Figure 2e). The first group mainly included studies on apiary location [57], beekeeping suitability [58], spatial honey production potential [16], and hive management or planning [59], where the target bee taxon was not specified. The second group included studies centered on *A. mellifera*, including colony health [60], foraging behavior [61], honey yield and colony productivity [62], and landscape interactions [43]. Beyond managed honey bee systems, 46 studies focused on other wild bee taxa, excluding *Bombus* spp. and Meliponini, and addressed diversity patterns [63], habitat suitability [64], land-use effects [65], and conservation planning [66]. Bee-associated organisms were also analyzed in 28 studies, including pests, pathogens, parasites, and predators such as *Varroa destructor* [67], *Aethina tumida* [56], *Paenibacillus larvae* [68], *Vespa velutina* [69], and *Palarus latifrons* [19]. Studies on *Bombus* spp. (n = 24), stingless bees/Meliponini (n = 11), and other *Apis* spp. (n = 4) were less frequent but showed the use of GIS in bumblebee habitat and community studies [40,45,70], meliponiculture and stingless bee conservation [71,72], and other *Apis* species distribution or habitat studies [73,74], respectively.

### 3.2. Bibliometrics of Publications

#### 3.2.1. Countries and Institutions Co-Authorship

**Figure 3:**
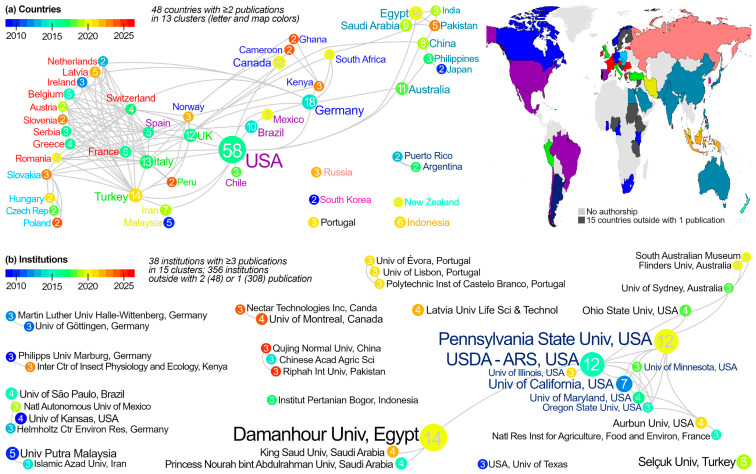
Bibliometric network of co-authorship among countries (**a**) and institutions (**b**), showing the number of publications (number and size of circles), the average publication year (circle color), and collaborative clusters (label color and spatial grouping).

A total of 63 countries contributed to bee publications using GIS tools, of which 20 countries had five or more publications (Figure 3a). The US (n = 58) was the leading author country, with more than three times the publications of Germany (n = 18), the second most productive country, followed by Egypt and Turkey (n = 14 each). The collaboration network included 48 countries (≥2 publications) organized into 13 clusters, of which seven were interconnected and six remained isolated, notably Indonesia (n = 6) as the most productive isolated country. The cooperation networks emerged mainly between neighboring countries, and the largest cluster (C1, 11 countries) grouped predominantly European countries, while C2 (8 countries) reflected research ties between Egypt, Saudi Arabia, and Asia-Pacific countries. C3 (7 countries) connected Germany with African and North American countries, and C4 (5 countries) linked the US with Latin American nations and Spain. Regarding temporal patterns, Germany, Brazil, and the United Kingdom (UK) showed earlier average publication years (2014–2016), followed by the US and Italy (2017), whereas Canada, Egypt, and Turkey showed more recent average publication years (2020–2021).

A total of 398 institutions contributed to the 228 reviewed publications, of which 38 had three or more publications (Figure 3b). The leading institution by number of publications was Damanhour University, Egypt (n = 14), followed by Pennsylvania State University and USDA—ARS (Agricultural Research Service), both in the US (n = 12 each), the University of California, US (n = 7), and Selçuk University, Turkey (n = 6). The institutional network was fragmented into 15 clusters, of which only four were interconnected, while 11 remained isolated. The clearest same-country grouping was observed among US institutions, whereas smaller groupings appeared among institutions from Germany, Australia, and Portugal. Cross-country links were also observed, such as Egypt–Saudi Arabia and Malaysia–Iran. Earlier average publication years were observed for the University of Texas, Universiti Putra Malaysia, the University of Göttingen, and the University of California (2010–2012), whereas more recent average years were observed for Damanhour University, Pennsylvania State University, Latvia University of Life Sciences and Technologies, Nectar Technologies Inc., and the University of Montreal (2020–2025).

#### 3.2.2. Authors and Their Keywords

**Figure 4:**
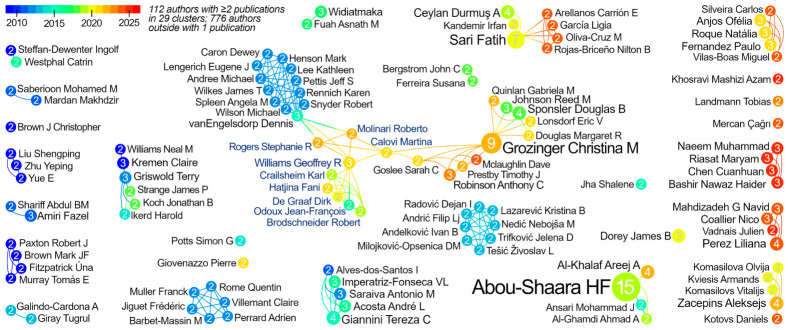
Temporal co-authorship clusters and number of publications per author.

A total of 888 authors contributed to publications using GIS tools in bee research, of which 112 authors had two or more publications (Figure 4). Abou-Shaara HF was the leading author by number of publications (n = 15), followed by Grozinger Christina M (n = 9) and Sari Fatih (n = 7). Six authors contributed four publications, 21 authors contributed three, and 82 authors contributed two, while 776 authors published only one paper. This highly skewed productivity pattern was consistent with Lotka’s Law, both for the theoretical model (β = 2; *p* = 0.203) and the fitted model (β = 2.73; *p* = 0.938). The collaborative network of authors with at least two publications was organized into 29 clusters, mostly small and weakly connected (Figure 4). This pattern indicated that recurrent collaboration was concentrated in specialized groups rather than forming a single cohesive author community. More recent average publication years were observed in clusters led by Abou-Shaara HF and Sari Fatih (average publication year = 2020), mainly associated with beekeeping suitability and apiary-location modeling [75,76,77,78]; Grozinger Christina M (average publication year = 2022), associated with spatial risk assessment and decision-support tools for pollinator health [79,80]; Zacepins Aleksejs (average publication year = 2022), associated with precision beekeeping and apiary scheduling [59,81,82,83]; and Pérez Liliana (average publication year = 2025), associated with machine-learning and remote-sensing approaches for beekeeping suitability, crop mapping, and hive survival analysis [54,84,85].

**Figure 5:**
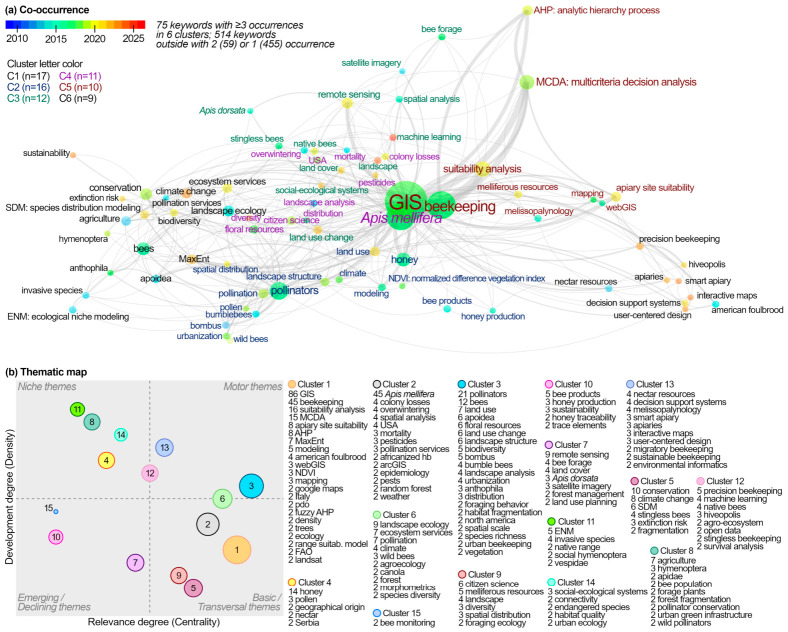
Author keyword structure in GIS-related bee research, showing temporal co-occurrence patterns among keywords with at least three occurrences (**a**) and thematic clusters based on Callon’s centrality and density for keywords with at least two occurrences (**b**).

A total of 589 author keywords were identified, of which 75 had three or more occurrences and were included in the co-occurrence network (Figure 5a). The most frequent keywords were ‘GIS’ (n = 86), ‘beekeeping’ (n = 45), ‘*Apis mellifera*’ (n = 45), ‘pollinators’ (n = 21), ‘suitability analysis’ (n = 16), and ‘multicriteria decision analysis’ (MCDA, n = 15). The co-occurrence network was organized into six clusters. C5, the cluster with the highest cumulative number of occurrences, linked ‘GIS’, ‘beekeeping’, ‘suitability analysis’, ‘multicriteria decision analysis’ (MCDA), ‘Analytical Hierarchy Process’ (AHP), and ‘apiary site suitability’, reflecting the strong concentration on suitability-based apicultural applications. C1 grouped broader ecological and biogeographic terms, including ‘bees’, ‘landscape ecology’, ‘climate change’, ‘species distribution modeling’ (SDM), ‘MaxEnt’, and ‘conservation’. C2 connected ‘pollinators’, ‘wild bees’, ‘bumble bees’, ‘land use’, ‘landscape structure’, and ‘urbanization’, indicating a landscape-ecology focus on pollinator communities. C4 was centered on ‘*Apis mellifera*’ and included ‘colony losses’, ‘mortality’, ‘overwintering’, and ‘pesticides’. C3 combined ‘remote sensing’, ‘satellite imagery’, ‘spatial analysis’, ‘land cover’, and ‘bee forage’, whereas C6 grouped terms related to decision-support and precision beekeeping, including ‘decision support systems’, ‘precision beekeeping’, ‘smart apiary’, ‘interactive maps’, and ‘hiveopolis’. Temporally, recent keywords in the co-occurrence network included ‘machine learning’ (2025), ‘smart apiary’ and ‘interactive maps’ (2024), ‘climate change’ and ‘precision beekeeping’ (2023), and ‘MaxEnt’ (2022).

The thematic map of the 134 author keywords with two or more occurrences identified 15 clusters and classified them according to Callon’s density and centrality as motor themes (high density and centrality), niche themes (low centrality and high density), emerging or declining themes (low density and centrality), and basic or transversal themes (high centrality and low density) (Figure 5b). The basic and transversal structure of the field was dominated by the cluster centered on ‘GIS’, ‘beekeeping’, ‘suitability analysis’, MCDA, and AHP, confirming the central role of GIS-based suitability analysis in apicultural studies. Motor themes were mainly associated with pollinator ecology, floral resources, land use, biodiversity, landscape ecology, ecosystem services, and pollination, showing the consolidation of landscape-oriented ecological applications. Niche themes included more specialized topics such as ecological niche modeling, invasive species, nectar resources, decision-support systems, and precision beekeeping. Emerging or declining themes were represented by clusters related to conservation, climate change, species distribution modeling, extinction risk, remote sensing, bee forage, and land cover.

#### 3.2.3. Core Sources

**Figure 6:**
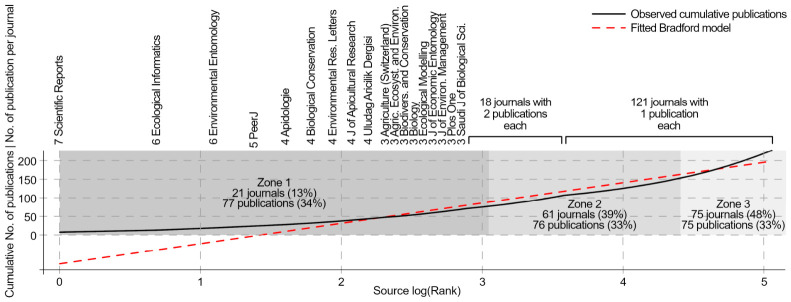
Core sources and Bradford zones for publications on GIS applications in bee research.

The 228 publications were distributed across 157 sources, showing a dispersed publication pattern. Bradford’s law identified 21 core sources that concentrated 77 publications, corresponding to approximately one-third of the reviewed corpus (Figure 6). The most productive source was Scientific Reports (n = 7), followed by Ecological Informatics and Environmental Entomology (n = 6 each), and PeerJ (n = 5). However, 121 sources contributed only one publication, indicating that GIS-related bee research was distributed across a broad set of publication outlets. The Kolmogorov–Smirnov test indicated a significant deviation from the theoretical Bradford distribution (D = 0.191, *p* = 0.006), supporting this dispersed publication pattern.

### 3.3. GIS Analysis Themes and Tools

**Figure 7:**
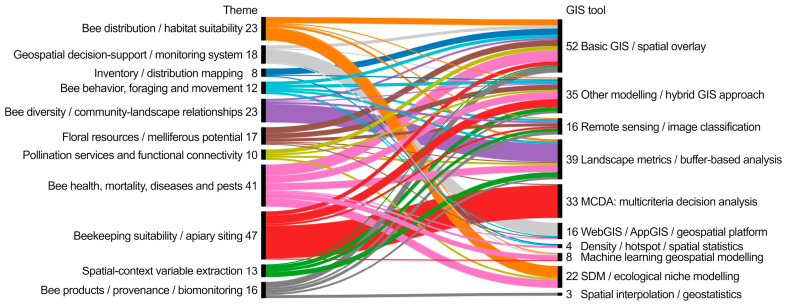
Relationship between GIS application themes and main GIS tools used in bee research.

GIS was mainly used to assess land suitability for beekeeping and apiary siting (n = 47, 21%) and to analyze bee health, mortality, diseases, and pests (n = 41, 18%) (Figure 7). Other frequent themes included bee distribution/habitat suitability and bee diversity/community–landscape relationships (n = 23 each, 10%), followed by geospatial decision-support or monitoring systems (n = 18, 8%), floral resources/melliferous potential (n = 17, 8%), and bee products/provenance/biomonitoring (n = 16, 7%). Regarding tools, basic GIS/spatial overlay was the most used approach (n = 52, 23%), followed by landscape metrics/spatial context analysis (n = 39, 17%), other modeling or hybrid GIS approaches (n = 35, 15%), MCDA (n = 33, 15%), and species distribution modeling/ecological niche modeling (n = 22, 10%). These themes and tools are addressed in the following subsections.

#### 3.3.1. Land Suitability for Beekeeping and Apiary Location

**Figure 8:**
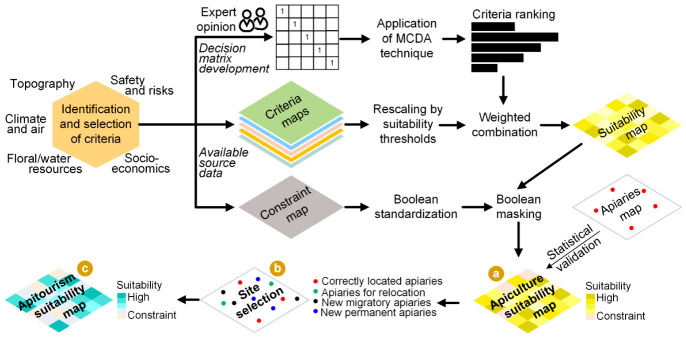
Conceptual model of MCDA in GIS with a beekeeping approach.

Of the 47 studies on land suitability for beekeeping and apiary siting, 32 used MCDA-GIS as the main tool. Malczewski and Rinner [86] provide a comprehensive description of the theories, methods, and technologies for integrating MCDA into GIS. For beekeeping, in summary, (i) the criteria that determine beekeeping activity are identified and selected, such as floral resources, proximity to water sources, land use, topography, and climatic conditions; (ii) these criteria are mapped using remote sensing and GIS to create spatial layers that represent each criterion and are weighted by experts using MCDA techniques according to their relative importance for beekeeping; (iii) the criteria maps, reclassified into categories according to suitability thresholds, are combined using weighted overlay to produce a composite suitability map (Figure 8—point a); and (iv) the final suitability map is validated against maps of existing apiaries and fieldwork, allowing the spatial agreement between predicted suitability and real apiary locations to be assessed (Figure 8—point b). This process highlights the strength of MCDA in integrating diverse datasets and expert judgment into a spatial framework, allowing for nuanced and data-driven decision-making.

A total of 30 criteria were used in the MCDA for the apicultural suitability of the territory, which were grouped into five categories (Table 1). The justification for each criterion is explained in Sari et al. [3,75] and beekeeping manuals. It is also common to mask the final suitability map with a constraint map, such as rivers, roads, and urban areas [87], to avoid overestimating suitable areas. Among the MCDA techniques, the Analytical Hierarchy Process (AHP) was the most widely used to weight the criteria (24/32 studies, 75%). The remaining studies either did not weight the criteria or used other approaches, including Technique for Order Preference and Similarity to Ideal Solution (TOPSIS), Preference Ranking Organization Method for Enrichment Evaluations (PROMETHEE), Fuzzy AHP, and Fuzzy logic/overlay. The conceptual model of these and other MCDA techniques is described in detail in Thakkar [88]. MCDA techniques have also been compared to assess the suitability of the territory for beekeeping; for example, AHP, TOPSIS and VIKOR (Vise Kriterijumska Optimizacija I Kompromisno Resenje) were compared in [3], AHP and PROMETHEE in [75], AHP and Full Consistency Method (FUCOM) in [44], and Fuzzy AHP and Fuzzy overlay in [89].

**Figure 9:**
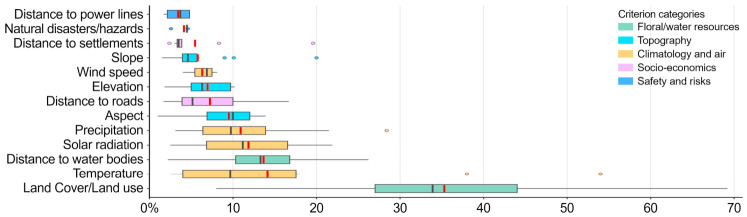
Boxplot of importance weights of criteria reported in at least three AHP studies, where red lines indicate medians, black whiskers indicate variability, and dots indicate outliers.

In the 24 AHP studies identified, the floral resources stood out as the most frequently used and highest-weighted criterion (Figure 9). Floral resources were mainly represented by land cover/land use (LULC) or ecosystem maps, where natural vegetation cover or specific crops were valued as more suitable for beekeeping. The LULC maps used in MCDA were mainly obtained from secondary sources, such as global or national maps, and only in some cases were generated by satellite image classification at local scales (see Section 3.3.2). Criteria related to the distance to plant polygons of beekeeping interest within the LULC map were also used [102]. One approach to improve MCDA was to consider the flowering seasonality of plants of beekeeping interest within the LULC map [103,104]. The suitability of the territory for plants of beekeeping interest was also evaluated by MCDA [105]. MCDA was also used to evaluate future apicultural suitability, either by predicting the LULC variable with a CA-Markov model [101] or by using predicted temperature and precipitation variables [58,77].

MCDA studies at national or subnational geographic scales mainly focused on territorial suitability for *A. mellifera*, although some studies focused on meliponiculture [71,72]. At the local scale, particularly in areas influenced by civil projects, a method was recently proposed to assess the suitability for *Bombus affinis* [106]. In addition, some studies sought to integrate beekeeping with other economic activities, such as tourism through apitourism [97] (Figure 8—point c). Combining a beekeeping suitability map with the suitability map of another activity could support sustainable complementary activities. After obtaining an apicultural suitability map through MCDA, it is also possible to calculate the melliferous potential of the most suitable areas [16], for which a map of melliferous vegetation is required (see Section 3.3.2).

#### 3.3.2. Remote Mapping of Floral Resources and Productivity Potential

**Figure 10:**
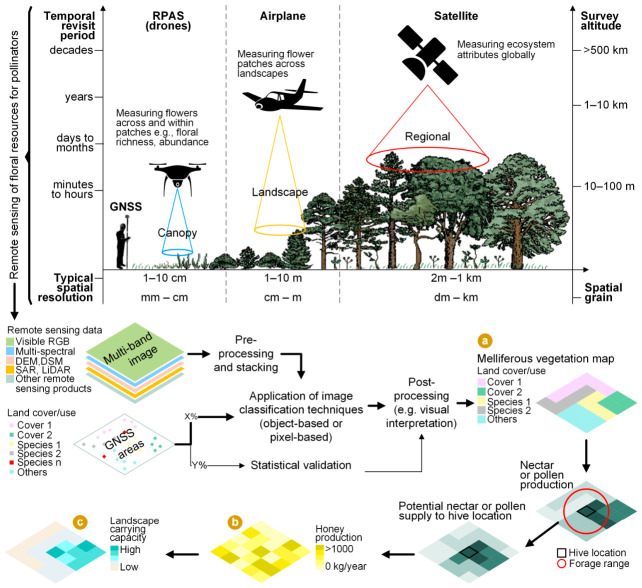
Conceptual model of remote sensing of floral resources for pollinators.

Of the 228 reviewed studies, 17 (8%) focused on mapping areas with floral resources of interest to bees. For this purpose, MCDA (see Section 3.3.1), SDM (see Section 3.3.4), and remote sensing were used, with remote sensing being the main approach. Chuvieco [107] provides a complete description of the fundamentals of satellite remote sensing and Google Earth Engine (GEE) serves as a platform for accessing multitemporal satellite imagery while also providing tools for processing such data, which has gained significant reach and application in recent years [108]. In summary, (i) specific land cover or plant mosaics of interest (points, lines, or polygons) are georeferenced using GNSS (Global Navigation Satellite System) technology to create reference datasets; (ii) landscape data are collected using different remote sensing platforms (satellites, airplanes, or drones) and sensors (passive and active), and multiband images are constructed to capture spectral information across various wavelengths; (iii) using the spectral signatures and/or unique spectral shapes of the georeferenced mosaics, together with image classification techniques such as object-based or pixel-based methods, thematic landscape maps are generated to categorize land cover types; and (iv) these maps are validated by comparison with additional georeferenced mosaics to assess accuracy (Figure 10—point a).

The resulting thematic landscape maps assign each pixel of the remote sensing images to a LULC type (Figure 10—point a), from which the floral resource of interest, or melliferous vegetation, is commonly extracted as a criterion for analyzing territorial suitability for beekeeping [17,109]. Floral resources can be mapped as general mosaics of forested or agricultural areas, or as specific plant species of interest [73,110]. A recent review addressed in greater detail the remote sensing of floral resources for pollinators, including satellite data and new opportunities provided by Remotely Piloted Aircraft Systems (RPAS or drones) [111]. Another review addressed trends in tree classification and segmentation using RPAS data, with emphasis on agroforestry systems [112].

Honey production potential can be calculated from floral resource maps (Figure 10—point b), based on the theoretical amount of nectar and pollen that can be generated by each mapped plant within each analysis pixel. The most rigorous approach was based on estimating nectar production per flower and per plant for each species and then summing these amounts at the plant community and land-cover type levels [113]. A given pixel can then be considered as a hive location, and the potential supply of nectar or pollen from nearby pixels within the bee foraging range can be calculated. In Italy, a 3 km bee foraging area around each pixel of honey vegetation was considered, and honey production maps (kg/year) were obtained [16]. Because theoretical values of plant nectar and pollen are not always available, an expert-based hedge prioritization score can be assigned, as was done in New Zealand [114]. In addition, the relationship between potential production and theoretical hive demand can be used to infer a potential carrying capacity of the landscape to sustain hives monthly throughout a full year [115] (Figure 10—point c).

Based on LULC data derived from remote sensing, an indicator of the pollination potential of wild solitary bees was also developed throughout Germany [46]. In Latvia, remote sensing images of agricultural fields were used to develop a model for selecting suitable apiary locations [81]. In the first step, fields in the aerial image of the region were annotated as polygons, and an estimated resource value was assigned to each field, resulting in a semantically annotated map. In the second step, the method calculated a value function by assigning to each location on the map an estimated amount of resources available for collection. This model was later improved by proposing a system capable of identifying the ideal number of bee colonies needed for optimal foraging [82]. The improved model considered multiple factors, such as the number of fields in the area, field productivity, potential contamination level, and the presence of infrastructure.

#### 3.3.3. Extraction and Visualization of Information

**Figure 11:**
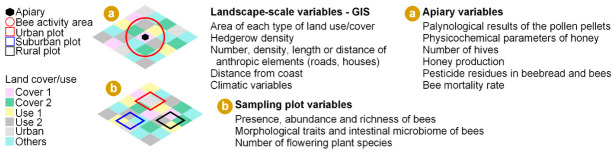
Conceptual model of information extraction in GIS with a bee analysis approach.

Of the 228 reviewed studies, landscape/context data extraction (n = 13, 6%) and inventory/distribution mapping (n = 8, 4%) were identified as specific GIS application themes, commonly supported by basic GIS tools. In summary, (i) parameters of a local environment, such as an apiary or sampling plot, were analyzed; (ii) parameters of the surrounding landscape were extracted using GIS; and (iii) both groups of parameters were statistically compared for different purposes outside GIS, such as assessing correlations, predicting outcomes (e.g., honey production potential), or evaluating the effects of landscape features on bee diversity, morphology, and performance (Figure 11). When the local environment was an apiary (Figure 11—point a), studies analyzed physicochemical parameters of honey [116], pollen granules through palynological analysis [117], or pesticide residues in pollen, honey, beebread, beeswax, and bees [7,118,119]. These parameters were interpreted using landscape variables extracted around the apiary and within the bee activity area. From global or national LULC maps, the most frequently extracted landscape variable was the percentage of area of each LULC class within the bee activity area. A method for predicting the potential honey production of an apiary as a function of landscape LULC was also proposed [120]. In addition, the effect of spring–summer seasonal changes in floral resources (LULC) on colony performance was evaluated [121].

When the local environment was a sampling plot (Figure 11—point b), studies assessed how landscape characteristics, mainly human disturbance, influenced bee diversity [42] or morphology [122]. Urban-focused studies examined the percentage of impervious surfaces, such as roads, buildings, parking areas, or similar structures [11], as well as the amount, density, length, or distance to anthropogenic elements, including roads, houses, and others. In addition to extracting data from LULC maps, other studies extracted additional variables, such as the heterogeneity index and the ecosystem integrity index [50], or climatic variables [123,124]. For information extraction studies, the analysis radii varied between 0.4 km [125] and 4.8 km [126], depending on the bee species of interest or the purpose of the study, with 1, 2 or 3 km radii being the most common.

Maps played a fundamental role in the spatial representation of information. Tabular information represented on maps acquired a greater geographic context. The reviewed studies mapped different variables related to bees, including apiary count or density by administrative units [127], spatial and temporal trends in the economic value of pollination services [1], national survey reports of winter colony losses [128,129], and total honey production [130]. The first two studies identified in this review visually represented the migration processes of Africanized honey bees in the Americas [35,131].

#### 3.3.4. Bee Diversity, Landscape Relationships, and Pollination Services

GIS was also used to relate bee diversity, community structure, and pollination services to landscape composition and configuration. In community-level studies, landscape metrics and spatial-context variables were used to evaluate how forest cover, agricultural land, urbanization, habitat loss, edge effects, and conservation interventions influenced bee richness, abundance, or assemblage composition [63,65,132,133,134]. These approaches were applied to different ecological contexts, including forested ecosystems, tropical deforestation gradients, remnant prairies, agricultural landscapes, and conservation-intervention areas. Other studies focused on pollination services and functional connectivity, using GIS to estimate crop pollination value, identify areas important for maintaining pollination services, or evaluate habitat connectivity for pollinators [1,126,135,136,137]. In these cases, the spatial analysis was not centered on bee movement itself, but on how landscape structure supported pollination functions, crop production, or conservation planning.

#### 3.3.5. Distribution Models of Bee Species, Pests, and Plants

**Figure 12:**
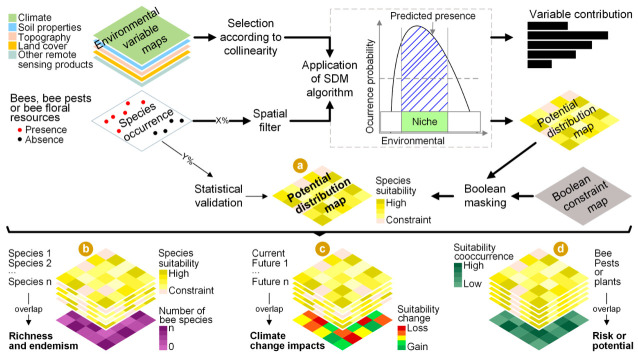
Species distribution models (SDMs) conceptual model in GIS for bee surveys.

Of the 228 studies, 22 (10%) used species distribution models (SDMs) or ecological niche modeling (ENM) in GIS. SDMs and ENMs, based on statistical and cartographic protocols, combine observed species presence or presence/absence data with environmental variables to estimate the potential distribution of a given species [138]. Franklin [139] provides a comprehensive description of the theories and methods for mapping species distributions. In summary, (i) a database of georeferenced occurrence records of the species of interest is constructed and spatially filtered to reduce sampling bias and ensure even representation; (ii) the environmental variables that limit the distribution of that species are mapped and selected to reduce collinearity; (iii) SDM or ENM algorithms are applied to predict maps of current and future environmentally suitable habitats, often under multiple climate scenarios, to assess potential shifts in species distribution; and (iv) the resulting distribution maps are validated using independent occurrence records or partitioned datasets to evaluate their predictive accuracy and reliability (Figure 12—point a). This iterative process ensures that the models capture the ecological requirements of the species while accounting for spatial and temporal variability.

As input, SDMs require georeferenced observations of the species of interest and geographic layers of environmental information, which are now widely available in digital format. Online repositories that provide species observation data include the Global Biodiversity Information Facility (GBIF, https://www.gbif.org/, accessed on 27 April 2026), iNaturalist (https://www.inaturalist.org/, accessed on 27 April 2026), Tropicos (https://www.tropicos.org/home, accessed on 27 April 2026), and various digital herbaria. Climate data can be obtained from sources such as WorldClim [140], CHELSA (Climatologies at high resolution for the earth’s land surface areas) [141], and ENVIREM (ENVIronmental Rasters for Ecological Modeling) [142]. For topography, commonly used datasets include SRTM (Shuttle Radar Topography Mission), ALOS/PALSAR (Advanced Land Observation Satellite/Phase Array type L-band Synthetic Aperture Radar), and ASTER (Advanced Spaceborne Thermal Emission and Reflection Radiometer) [143]. Soil properties, particularly useful for plant-related SDM, are available through SoilGrids [144]. Land cover and other remote sensing products are accessible via the GEE platform [108]. Species observations at specific sites are analyzed alongside prevailing environmental conditions using statistical and machine-learning algorithms, enabling the generation of potential species distribution maps across space and time.

The maps predicted with SDMs were generated for bee species [2], bee pests or predators [145,146], and plants of interest to bees [147] (Figure 12—point a). Multi-species SDMs can be stacked to identify areas of bee species richness or endemism [2] (Figure 12—point b). An innovative tool developed for this purpose is SSDM, an R package for stacked species distribution modeling [148]. SDMs were assessed under current climate conditions or projected to future scenarios, mainly using future climate variables, to determine the risk of habitat loss under climate change [10,47,135] (Figure 12—point c). Potential bee distribution maps were also overlaid with pest maps or floral resource maps to determine areas of current and future co-occurrence (Figure 12—point d). Recent applications also combined habitat suitability modeling with remote sensing and structural equation modeling to assess how land-use/land-cover change, NDVI, and greenhouse gas emissions affected bumblebee habitat suitability. The most commonly used SDM algorithm in the reviewed studies was the Maximum Entropy approach [149]; however, one study recommended considering other algorithms, such as Classification and Regression Tree [150]. SSDM offers a range of algorithms, methodological approaches, and parameterization options for each step of the SDM construction process [148].

#### 3.3.6. Bee Behaviors, Foraging, and Movement

Some bee behaviors were analyzed and tracked using GIS. The spatial distribution of migratory *A. mellifera* swarms and beekeeping accidents was mapped [151,152]. GIS and remote sensing were used to help locate drone congregation areas [48], while MCDA was applied in GIS to identify suitable areas for controlled honey bee mating in Italy [153]. The honey bee waggle dance was also analyzed to produce a spatial probability distribution of the resource location communicated by the dance [18]. In addition, a visual analytics system developed in Australia allowed users to analyze bee drift data using HoloLens as a head-mounted augmented reality interface [154].

#### 3.3.7. Bee Health, Diseases, Pests, and Mortality

Density analysis allowed the identification of areas at risk of demographic decline [9], while landscape-based approaches examined relationships between spatially distributed winter mortality rates, environmental and biological conditions, and apiary management [155]. At national and regional scales, several studies mapped honey bee colony losses or winter mortality and related them to climatic, biological, management, or landscape variables [128,129,155,156]. Disease-related applications included the spatial monitoring of *Varroa destructor* infestation in apiaries [67], American foulbrood infection pressure using Google Maps [68], the distribution of apiary density and bacterial brood diseases in Switzerland [127], and the geographic distribution of *Paenibacillus larvae* isolates in New Zealand [157]. Other studies focused on pests, predators, or invasive species that threaten bees, such as *Aethina tumida* [56], *Vespa velutina* [158], *Palarus latifrons* [19], and *Megaselia scalaris* [145], mostly using species distribution modeling or spread-risk approaches. GIS was also applied to pesticide-related mortality and risk assessment, including bumble bee mass mortality caused by neonicotinoids [159] and county-level insecticide hazard to honey bees [80]. More recent studies combined landscape, management, weather, and health variables to explain viral loads or predict hive survival using statistical or machine-learning approaches [54,60,84,160].

#### 3.3.8. Bee Products, Biomonitoring, and Traceability

Spatial interpolation was used to monitor the distribution of different parameters in bee products and bee matrices. Jin and Heap [161] provide a complete description of spatial interpolation methods in GIS. In summary, (i) point values of a parameter of interest are collected; (ii) exploratory data analysis is performed, including normality, trends, autocorrelation, and semivariogram analysis, and a spatial interpolation method is applied to predict a continuous map of the parameter of interest; (iii) depending on the method, explanatory variables with low collinearity can be incorporated; and (iv) the continuous maps are validated with additional point values. GIS was also used to map honey value-chain routes and final market destinations, as shown for honey commercialization from the Amazonas region of Peru [162].

Honey and bee matrices were analyzed to model continuous maps of the spatial distribution of heavy metals and metalloids in Manchester (UK) [8], and trace element concentrations and lead isotopic compositions in Vancouver (Canada) [163]. At the regional scale, studies in Serbia [164,165] and India [166] used spatial interpolation in GIS for geographic-origin recognition and for mapping distribution patterns of chemical and palynological traits of multifloral honey. In Nigeria, spatial interpolation mapping showed variations in physicochemical and bioactive compounds in honey and identified geographical locations suitable for the production of alkaloid-rich anti-enteric honey, a marker associated with activity against resistant enteric bacilli [167].

#### 3.3.9. Geospatial Decision-Support and WebGIS Platforms

WebGIS platforms were also designed for precise tracking and traceability services using GNSS technology, including beehive locations and bee-product quality, with applications reported in Greece [168], Italy [169], Spain [170], and China [171,172]. Health-related decision-support tools were also developed, such as a Google Maps-based AppGIS in the Czech Republic for spatial problems associated with American foulbrood disease [68]. More recent WebGIS and interactive platforms expanded toward beekeeping planning and monitoring, including Google My Maps for healthy apiary location in the Philippines [173], a WebGIS-based interactive map in Latvia for scheduling beekeeping activities through flowering calendars, weather information, plant information, and links to remote hive monitoring data [59], and OpenStreetMap-based environmental assessment for precision beekeeping [83]. Other applications included WebGIS for member distribution and honey sales management in Indonesia [174], Beescape/Beescape NexGen as spatial decision-support systems for pollinator health and beekeeping decisions [79,175,176], a system for recommending native stingless bee species for meliponiculture [177], and a web-based geospatial atlas of honey bee forage plants [52]. A recent review addressed remote beehive monitoring in greater detail under the concept of precision beekeeping [24], emphasizing the importance of integrating GIS and GNSS technologies for accurate monitoring of beekeeping variables.

#### 3.3.10. Bees in Urban Environments

Urban contexts were included in 45 studies (20%), including studies with an explicit urban focus, urban–rural gradients, or urban variables used as explanatory covariates (Figure 2c). In addition to the urban studies mentioned above, Samuelson and Leadbeater [178] proposed a method to generate urban LULC maps at multiple biologically relevant foraging ranges around study sites to identify land-cover types relevant to pollinators. In Belgium, the connectivity of green roofs and their role in strengthening bee biodiversity and urban ecological networks were analyzed [39], while bee communities on Australian green roofs were recently assessed using local attributes and GIS-derived landscape variables [38]. A method combining Google Street View and geospatial video mapping was proposed in the USA to virtually inventory vegetation composition over time in residential yards and assess habitat for native pollinator conservation [49]. More recent urban analyses modeled green-space connectivity and pollinator habitat in New York City [66], pollen movement across urban landscape features in Raleigh [179], and urban beekeeping suitability and hive survival in Montreal [37].

#### 3.3.11. Other Spatial Analyses

In Japan, a geographic profiling approach was applied to predict the location of *Bombus terrestris* nests, assuming that feeding and nesting sites were analogous to crime sites and offenders’ residences, respectively [70]. In the UK, a process-based pollinator model was used to explore how solar park configuration and surrounding landscape management could improve ground-nesting bumblebee density and nest productivity [180]. In Ghana, the spatial incongruity between mining concessions, forest cover, and beekeeping activities was analyzed [181]. In Australia, the risk of extinction of more than 500 bee species caused by the impacts of catastrophic bushfires was modeled [182].

## 4. Discussion

### 4.1. General Interpretation of GIS Applications in Bee Research

Overall, the reviewed literature showed that GIS applications in bee research were structured around three complementary functions: spatial description, spatial explanation, and spatial decision support. First, GIS was used to organize and visualize spatially explicit information, such as occurrences, apiaries, bee products, diseases, and study sites. Second, GIS was used analytically to relate bee-related variables to landscape, environmental, climatic, or management conditions. Third, GIS supported applied decision-making through suitability maps, risk maps, monitoring platforms, and interactive tools. This distinction is important because the reviewed studies differed not only in the GIS tools applied, but also in the role that spatial analysis played in each study. In this sense, the classification proposed here separates the purpose of GIS use from the methodological tool, providing a clearer framework for interpreting GIS applications across apiculture, bee ecology, pollination services, health risk, and conservation studies.

The taxonomic structure of the reviewed studies also showed that GIS applications have been more strongly developed for managed beekeeping systems and *A. mellifera* than for other bee groups. This pattern reflects the economic and operational relevance of managed honey bees, especially for apiary siting, honey production, colony health, and beekeeping management. However, it also indicates that GIS-based research on other wild bees [63,65], *Bombus* spp. [40,70], stingless bees/Meliponini [71,72,182], and other *Apis* species [73,74] remains comparatively less developed. This imbalance is important because different bee groups differ in nesting habits, foraging ranges, habitat requirements, social organization, and sensitivity to landscape change. Therefore, GIS workflows designed for managed honey bees should not be directly generalized to wild bee communities without considering taxon-specific ecological traits. Expanding GIS applications to underrepresented bee groups would improve the ecological scope of the field and support more biologically explicit conservation and management strategies.

### 4.2. Methodological Limitations and Improvement of MCDA Workflows

Beekeepers usually choose apiary locations based on previous experience, and these locations are not always optimal for bee colonies [81]. MCDA techniques integrated into GIS stood out as the most widely used tools for evaluating territorial suitability for beekeeping and selecting apiary sites (Figure 8). In the reviewed studies, criteria were often weighted in a single comparison matrix, where all criteria were compared against each other. However, some studies first weighted groups of criteria by category and then weighted the categories themselves [92], which can improve expert consistency. A larger number of criteria compared simultaneously increases the risk of inconsistency [183]. Therefore, future studies should consider grouping criteria into categories and generating suitability submodels before producing the final suitability model.

In MCDA techniques, expert opinion strongly influences the results because criterion weights may be biased [86]. This limitation can be addressed by combining MCDA with data-driven approaches. SDMs, for example, provide information on the relative contribution of environmental variables used to model the potential distribution of a species of interest (Figure 12). These relative contributions could be used as data-driven importance weights in MCDA. Similar approaches have been applied to other economic activities, such as identifying suitable sites for aquaculture [184] and cocoa cultivation [185]. In beekeeping, future studies could use locations of highly productive apiaries as occurrence-like records in SDMs to identify the most influential environmental variables. Recent studies that compared weighting approaches, such as AHP with PROMETHEE [75] or AHP with FUCOM [44], also show the need to evaluate how different weighting methods affect suitability outputs.

The resulting MCDA maps usually subdivide the territory into suitability classes for beekeeping (Figure 8—point a), but they do not necessarily identify specific sites for planning an apiary network. For this purpose, a Near Analysis or other proximity-based GIS tools could be applied to the resulting MCDA map, as has been done in studies designing meteorological station networks [186]. Future research could therefore: (i) overlay the MCDA suitability map with existing apiaries to assess whether current apiary locations fall within suitable areas and, if necessary, relocate them to the nearest suitable polygons; (ii) identify new suitable apiary sites while respecting the foraging area of existing apiaries; and (iii) incorporate temporal or future suitability scenarios, such as land-use change or climate projections, to support long-term apiary planning.

### 4.3. Floral Resource Representation and Temporal Suitability in Beekeeping Models

The MCDA studies reviewed generally presented limitations when incorporating floral resources as a criterion of land suitability for beekeeping, since they often used general LULC maps as proxies for floral resources, considering vegetation cover as suitable and anthropogenic land uses as unsuitable. However, both the spatial distribution and temporal flowering phenology of key wild and agricultural species for beekeeping should be considered [103,104]. One promising approach is to stack SDMs of melliferous flora based on flowering phenology [187]. In this approach, distribution grids are stacked according to the monthly flowering period of each species to generate grids representing flowering species richness by cell and month of the year. This method was designed to model flowering forage availability and support migratory beekeeping systems. The approach can also be extended by projecting the same species under future climate scenarios to identify potential risk zones for floral resource availability [188].

### 4.4. Remote Sensing Opportunities for Mapping Floral Resources

Remote sensing offers strong potential for studying floral resources critical to pollinators, yet its application in pollination research remains limited. Most studies relied on coarse-grain satellite imagery (Figure 10), which may fail to capture fine-scale variations in floral abundance, diversity, and volumetric characteristics, particularly in complex natural and urban habitats. Although drones can capture high-resolution imagery of individual flowers and floral patches, their use in pollination research remains underutilized, especially for studying interactions between pollinators and floral resources at fine spatial and temporal scales [111]. Hyperspectral remote sensing, which can detect subtle spectral variation associated with floral diversity and vegetation condition, also holds promise but remains underexplored. Addressing these gaps through drones, hyperspectral sensors, and improved classification methods could strengthen the mapping of floral resources, the analysis of pollinator behaviors, and the modeling of habitat requirements with greater spatial and temporal precision.

### 4.5. Reproducibility, FAIR Data, and Standardization Needs

The increasing use of GIS in bee research requires greater attention to data transparency, standardization, and reproducibility. The FAIR principles (Findable, Accessible, Interoperable, and Reusable) provide a useful framework for improving the management and reuse of scientific data [189]. In GIS-based bee studies, this implies reporting spatial data sources, coordinate reference systems, spatial resolution, buffer distances, classification rules, MCDA weights, validation datasets, and model settings. This is especially relevant for studies that generate reusable spatial datasets, occurrence databases, registries, or continental-scale mapping products. Examples from the reviewed corpus included occurrence datasets for *Anthidium* and *Bombus* species [190,191], national apiary registries used for spatial analysis [153], pesticide exposure datasets for pollinator research [80], and the geospatial atlas of honey bee forage plants [52]. Future studies should make spatial layers, scripts, criteria tables, occurrence records, and metadata available whenever possible, and should adopt standardized terminology for bee taxa, GIS tools, landscape variables, and cartographic products to improve comparability among regions, species, and beekeeping systems.

### 4.6. Emerging Technologies and Future Research Agenda

Recent studies showed a transition from static GIS mapping toward predictive, interactive, and data-driven spatial systems. Machine-learning approaches were used to classify pollination-relevant crops from Sentinel-2 time series [85], predict beehive survival and beekeeping suitability [37,54], and assess foraging landscape quality in commercial beekeeping using remote sensing and survival analysis [84]. Other recent applications incorporated artificial intelligence (AI), the Internet of Things (IoT), edge computing, and multimodal data for autonomous bee-health monitoring and hive-site optimization [60] or used OpenStreetMap and WebGIS environments for macro-level environmental assessment in precision beekeeping [83]. Interactive GIS tools also expanded toward operational scheduling, including flowering calendars and remote hive-monitoring links [59]. Future research should strengthen the integration of cloud-based GIS, machine learning, remote sensing, GNSS hive locations, smart-hive sensor data, and field floral inventories to model forage availability, colony stress, and apiary relocation needs at finer spatial and temporal scales. Emerging terms such as ‘deep learning’ and ‘geospatial artificial intelligence’ appeared only once, both in the Sentinel-2 crop-mapping study for beekeeping and pollination services [85], indicating that GeoAI-based approaches remain incipient in GIS-related bee research.

### 4.7. Limitations of This Review

This review identified publications that included the terms ‘bee’ and ‘GIS’ or their variants in the title, abstract, and keywords. Therefore, some bee-related studies that performed spatial analyses but did not explicitly mention GIS or equivalent geospatial terms in these fields may have been excluded. Despite this limitation, the strategy allowed us to focus on studies in which GIS or related spatial approaches were central or explicitly emphasized. Another limitation was the heterogeneity in how studies reported spatial methods, input data, criteria, weights, validation procedures, and cartographic outputs, which sometimes limited direct comparison among applications. In addition, some studies combined several purposes and tools, so the classification of a primary theme and main GIS tool necessarily simplified multifunctional studies. Future reviews could expand the search strategy by incorporating the most frequent GIS-related tools identified here, such as ‘MCDA’, ‘remote sensing’, ‘SDM’, ‘MaxEnt’, ‘WebGIS’, ‘machine learning’, and ‘spatial interpolation’, in combination with bee-related terms, to identify additional studies that used spatial analysis without explicitly referring to GIS.

## 5. Conclusions

Between 1977 and April 2026, this review identified 228 publications applying GIS and related geospatial approaches to bee research. Publication activity expanded markedly from 2000 to 2025, with a CAGR of 14.42%, showing the growing relevance of spatial analysis in this field. The reviewed literature was organized into 11 GIS application themes and 10 methodological tool families, highlighting that GIS has moved beyond simple mapping toward suitability modeling, landscape analysis, disease and pest risk assessment, floral resource mapping, biomonitoring, and decision-support systems. The most recurrent applications were beekeeping suitability and apiary siting, bee health and pest-related analyses, bee distribution and habitat suitability, and bee diversity–landscape relationships. The main methodological approaches included basic GIS/spatial overlay, landscape metrics, MCDA-GIS, remote sensing, species distribution modeling, WebGIS platforms, and emerging machine-learning geospatial models.

The synthesis also revealed important gaps. GIS-based bee research remains strongly oriented toward managed beekeeping systems and *Apis mellifera*, whereas wild bees, *Bombus* spp., stingless bees/Meliponini, and other *Apis* species are less represented. Future research should improve the temporal and taxonomic resolution of floral resource maps, integrate MCDA with data-driven models such as species distribution modeling, expand the use of remote sensing, GeoAI, and smart-hive data, and strengthen reproducibility through better reporting of spatial data, model settings, criteria weights, and validation procedures. Overall, GIS provides a flexible framework for linking bee ecology, apicultural management, environmental risks, and spatial decision-making, but its future value will depend on more standardized, reproducible, and biologically explicit applications.

## Figures and Tables

**Table 1:** Criteria used in the literature on MCDA-GIS for beekeeping.

Categories	Mapped Criteria	Selected References
Floral/water resources	Land cover, ecosystems, density and distance to agricultural and/or forestry plants, summer crops, NDVI (Normalized Difference Vegetation Index), quality level, density and distance to water bodies	[17,76,78,89,90,91]
Topography	Elevation, slope, aspect	[16,92,93]
Climatology and air	Precipitation, temperature, relative humidity, solar radiation, wind speed, level of air pollution	[72,76,94,95,96]
Socio-economics	Land use, distance to roads, railways, markets, buildings (urban areas) and settlements, tourism, protected natural areas	[75,97,98,99]
Safety and risks	Distance to power lines, natural disasters/hazards (landslides, forest fires, floods), mobile towers locations, genetically modified crops	[97,100,101]

## Data Availability

The data presented in this study are available on request from the author.

## Supplementary Materials

The following supporting information can be downloaded at: https://www.mdpi.com/article/10.3390/insects17060566/s1, Table S1: List of publications included in the review.

## Abbreviations

The following abbreviations are used in this manuscript:

AHP	Analytical hierarchy process
AI	Artificial intelligence
ALOS/PALSAR	Advanced land observation satellite/Phase array type l-band synthetic aperture radar
ASTER	Advanced spaceborne thermal emission and reflection radiometer
ARS	Agricultural Research Service
CHELSA	Climatologies at high resolution for the earth’s land surface areas
DEM	Digital elevation model
DSM	Digital surface model
ENVIREM	Environmental rasters for ecological modeling
FUCOM	Full Consistency Method
GEE	Google Earth Engine
GBIF	Global Biodiversity Information Facility
GIS	Geographic information system
GNSS	Global navigation satellite system
IoT	Internet of things
LULC	Land use/land cover
LiDAR	Light detection and ranging
MCDA	Multicriteria decision analysis
NDVI	Normalized Difference Vegetation Index
PROMETHEE	Preference ranking organization method for enrichment evaluations
RPAS	Remotely piloted aircraft system
SAR	Synthetic aperture radar
SDM	Species distribution models
SSDM	Stacked species distribution models
SRTM	Shuttle Radar Topography Mission
TOPSIS	Technique for order preference and similarity to ideal solution
UK	United Kingdom
US	United States
USDA	United States Department of Agriculture
VIKOR	Vise kriterijumska optimizacija i kompromisno resenje
