# Supplementary material for: Geographic Information System Applications in Bee Research
Source: Insects. 2026 May 29;17(6):566. doi: 10.3390/insects17060566 (PMC13299273; doi:10.3390/insects17060566)
Supplement: Supplementary file 1 [file insects-17-00566-s001.zip › insects-4235561-supplementary.pdf]

Review

Geographic Information System Applications in Bee Research

Nilton B. Rojas-Briceño [1,2,*], Jhonsy O. Silva-López [3,4], Betty K. Guzman [2,5], Manuel A. Ix-Balam [3], José L. Ramos-Tejeda [2], Manuel Oliva-Cruz [3], Jaris Veneros [3,*] and Ligia García [3]

Table S1: List of publications included in the review

Year	Authors	Title	Source title	DOI
2026	Khosravi Mashizi A.	Ecosystem Services And Disaster Risk Reduction Under Climate Change And Wildfire Threats	Environmental Management	10.1007/s00267-026-02405-4
2026	Tetlie J.; Dana C.; Thomas J.; Heads S.W.; Harmon-Threatt A.	Exploring The Impact Of Local Floral Quality And Land Use On Bumble Bee Communities: Insights Into Common And At-Risk Species	Biological Conservation	10.1016/j.biocon.2025.111648
2026	Naeem M.; Chen H.; Bashir N.H.; Wang H.; Riasat M.	Anthropogenic Land Conversion And Greenhouse Gas Emissions Drive A Decline In Bumblebee Habitat Suitability In Yunnan, China	Journal of Economic Entomology	10.1093/jee/toaf274
2026	Erwan; Muhsinin M.; Purnamasari D.K.; Sukarne; Supeno B.; Agussalim	Exploration Of The Natural Habitat Of Apis Dorsata Forest Bees In The Samota Biosphere Reserve, Sumbawa: An Ecological Study And Vegetation Diversity	HAYATI Journal of Biosciences	10.4308/hjb.33.3.716-728
2025	Landero-Hernández D.L.; del Carmen Ruiz-Acosta S.; de la Cruz M.Á.P.; Galindo-Alcántara A.	Productive And Health Diagnosis Of Beekeeping In The State Of Tabasco In The Period 2015-2018	Revista Mexicana De Ciencias Pecuarias	10.22319/rmcp.v16i4.6763
2025	Silveira C.; Roque N.; Leitão B.; Fernandez P.; Anjos O.; Vilas-Boas M.	Integrating Multicriteria Decision Analysis For Beekeeping Suitability: Insights From A Portuguese Honey Pdo Landscape	Earth Systems and Environment	10.1007/s41748-025-00860-3
2025	Mengistu F.; Hailu B.T.; Abera T.A.; Heiskanen J.; Zeleke T.T.; Johansson T.; Nkoba K.; Tuure J.; Pellikka P.	Climate-Smart Apiary Site Selection In East Africa: Gis-Based Analysis Of Current And Future Climate Projections	International Journal of Climate Change Strategies and Management	10.1108/IJCCSM-10-2024-0191
2025	Naeem M.; Rani A.; Lyu W.; Zhao H.; Riasat M.; Abbas S.; Hussain S.; Bashir N.H.; Li Q.; Chen H.	Temperature-Related Bioclimatic Variables Play A Greater Role In The Spatial Distribution Of Bumblebee Species In Northern Pakistan	Insects	10.3390/insects16010001
2025	Ojija F.; John J.P.	Anthropogenic Changes Influence Bee Populations, Floral Resource, And Land Cover Change In Mount Rungwe Nature Forest Reserve	CABI Agriculture and Bioscience	10.1079/ab.2025.0097
2025	Chen H.; Naeem M.; Meng L.; Bashir N.H.; Riasat M.; Liu Z.; Pan C.	Mapping Bumblebee Community Assemblages And Their Associated Drivers In Yunnan, China	Biology	10.3390/biology14091222
2025	Hung C.-C.; Hsueh C.-W.; Chang W.-C.; Yiin L.-M.	Associations Between Pesticides In Honeybees/Hive Products And Adjacent Land Use In A Rural Region Of Taiwan	Science of the Total Environment	10.1016/j.scitotenv.2025.179392
2025	Suhri A.G.M.I.	Saving Wallacetrigona Incisa: Community-Led Conservation Integrating Science And Indigenous Knowledge In North Luwu'S Mountain Forests, Indonesia	Jurnal Sylva Lestari	10.23960/jsl.v13i3.1165
2025	Mercan Ç.; Acibuca	Utilizing Fucom And Ahp Methods To Identify The Optimal Beekeeping Lands: A Case Study From Mardin, Türkiye	PLOS ONE	10.1371/journal.pone.0335784
2025	Mahdizadeh Gharakhanlou N.; Perez L.; Henry E.	Evaluating Environmental, Weather, And Management Influences For Sustainable Beekeeping In California And Quebec: Enhancing Beehive Survival Predictions	Journal of Environmental Management	10.1016/j.jenvman.2024.123783
2025	Godebo T.R.; Stoner H.; Taylor P.; Jeuland M.	Metals In Honey From Bees As A Proxy For Environmental Contamination In The United States	Environmental Pollution	10.1016/j.envpol.2024.125221
2025	Schiller J.; Brown J.; Hahs A.K.; Williams N.S.G.; Threlfall C.G.	Australian Green Roofs Attract Bees With Similar Functional Traits, But Species-Specific Responses To Local Attributes Differ Significantly	Basic and Applied Ecology	10.1016/j.baae.2025.06.008

2025	Lundquist M.J.; Lovejoy P.C.; Fay B.G.; Hernandez J.E.; Madrid M.	Bug Roads: Modeling The Green Space Connectivity And Pollinator Habitat In A Large City Using Open Gis Data And Tools	Ecological applications : a publication of the Ecological Society of America	10.1002/eap.70128
2025	Kotovs D.; Krievina A.; Zacepins A.	Enhancing Precision Beekeeping By The Macro-Level Environmental Analysis Of Crowdsourced Spatial Data	ISPRS International Journal of Geo-Information	10.3390/ijgi14020047
2025	Gómez-Fernández D.; García L.; Silva-López J.O.; Veneros Guevara J.; Arellanos Carrión E.; Salas-Lopez R.; Goñas M.; Atalaya-Marin N.; Oliva-Cruz M.; Rojas-Briceño N.B.	Suitability Of The Amazonas Region For Beekeeping And Its Future Distribution Under Climate Change Scenarios	Ecological Informatics	10.1016/j.ecoinf.2025.103082
2025	Laboisse S.; Vaillant M.; Cazenave C.; Kelečević B.; Chevalier I.; Andres L.	Space And Time Dynamics Of Honeybee (Apis Mellifera L.)-Melliferous Resource Interactions Within A Foraging Area: A Case Study In The Banja Luka Region (Bosnia & Herzegovina)	Biology	10.3390/biology14040422
2025	Kamnik R.	Assessing The Potential Of Urban Orchards, Berry Bushes, And Apiaries For Local Food Production And Carbon Mitigation In A Small European City	Challenges in Sustainability	10.56578/cis130208
2025	Roper O.I.; Youngsteadt E.	Bee-Mediated Pollen Transport Across Five Urban Landscape Features: Buildings Are Important Barriers	Ecology and Evolution	10.1002/ece3.71339
2025	Houtman L.; Robinson A.C.; McLaughlin D.; Grozinger C.M.	Evaluating The Usability And Utility Of A Spatial Decision Support System For Pollinator Ecology	Ecological Informatics	10.1016/j.ecoinf.2025.103182
2025	Nganso B.T.; Agboka K.M.; Atagong S.D.; Topé S.F.; Massing T.; Landmann T.; Sevgan S.; Mwiza W.; Odera F.; Piiru E.D.; Otieno-Ayayo Z.N.; Soroker V.; Guimapi R.A.	A Geospatial Atlas Of Honey Bee Forage Plants And Their Distribution Patterns In Africa And Beyond	Scientific Reports	10.1038/s41598-025-17240-6
2025	Marjanska E.; Moron D.	Habitat Composition Near Linear Landscape Structures Across Poland: Perspectives On Pollinator Conservation	PeerJ	10.7717/peerj.19765
2025	Nasser M.; Abou-Shaara H.; AlKhalaf A.A.; AlAshaal S.; Hosni E.	Modeling The Potential Range Expansion Of Hypotrigona Gribodoi (Stingless Bees) In Africa: Assessing Climate Change Impacts And Habitat Suitability Using Maxent And Gis	International Journal of Tropical Insect Science	10.1007/s42690-025-01555-y
2025	Kulow J.; Thiele J.; Westphal C.; Dauber J.	Landscape-Level Effects Of Local Conservation Interventions On The Abundance And Diversity Of Wild Bees In Agricultural Landscapes Of Germany	Biodiversity and Conservation	10.1007/s10531-025-03183-x
2025	Troshkov A.; Ermakova A.; Bogdanova S.; Ermakov I.	Algorithms And Technologies Of Geolocation Digitalization And Optimization Of Apiary Location	Lecture Notes in Networks and Systems	10.1007/978-3-031-98127-2_9
2025	Wanniarachchi P.W.A.S.V.; Ghanarathna K.M.P.M.; Vihansith W.G.P.; Wannigama S.V.; Chathumali C.; Siriwardana S.E.R.	Ai-Driven Autonomous Bee Health And Ecosystem Management System	ICAC 2025 - 7th International Conference on Advancements in Computing: The Future of Computing; AI, Quantum, and Beyond	10.1109/ICAC69156.2025.11361490
2025	Mahdizadeh Gharakhanlou N.; Vadnais J.; Perez L.; Coallier N.	Urban Buzz Or Urban Bust? Beekeeping Challenges, Suitability, And Survival Insights In Montreal, Canada	Ecological Informatics	10.1016/j.ecoinf.2025.103296
2025	Khosravi Mashizi A.; Escobedo F.	The Role Of Traditional Ecological Knowledge And Ecosystem Quality In Managing Ecosystem Services	Scientific Reports	10.1038/s41598-025-17611-z
2025	Malay A.; Weavers R.; Fedorka K.M.	Interactions Of Husbandry, Landscape, And Immunity In Regulating Viral Loads For Managed Honey Bees	Biology Open	10.1242/bio.062201
2025	Vadnais J.; Perez L.; Coallier N.	Assessing Foraging Landscape Quality In Quebec'S Commercial Beekeeping Through Remote Sensing, Machine Learning, And Survival Analysis	Journal of Environmental Management	10.1016/j.jenvman.2025.124157

2024	Rajagopalan K.; DeGrandi-Hoffman G.; Pruett M.; Jones V.P.; Corby-Harris V.; Pireaud J.; Curry R.; Hopkins B.; Northfield T.D.	Warmer Autumns And Winters Could Reduce Honey Bee Overwintering Survival With Potential Risks For Pollination Services	Scientific Reports	10.1038/s41598-024-55327-8
2024	Acosta A.L.; dos Santos C.F.; Imperatriz-Fonseca V.L.; Oliveira R.C.; Giannini T.C.	A Methodological Approach To Identify Priority Zones For Monitoring And Assessment Of Wild Bee Species Under Climate Change	Frontiers in Bee Science	10.3389/frbee.2024.1329844
2024	Fotso Kamga G.A.; Bouroubi Y.; Germain M.; Mbom A.M.; Chagnon M.	Expert Knowledge-Based Modelling Approach For Mapping Beekeeping Suitability Area	Ecological Informatics	10.1016/j.ecoinf.2024.102530
2024	Abou-Shaara H.F.; Sciberras R.	An Approach To Protected Designation Of Origin (Pdo) For Bee Honey Utilizing The Normalized Difference Vegetation Index (Ndvi)	Remote Sensing Letters	10.1080/2150704X.2024.2398816
2024	Abou-Shaara H.F.	Using Maximum Entropy Algorithm To Analyze Changes In The Distribution Of The Stingless Bees, Tetragonisca Angustula (Latreille, 1811), In Response To Future Climatic Conditions	Journal of the Entomological Research Society	10.51963/jers.v26i2.2567
2024	Landsman A.P.; Simanonok M.P.; Savoy-Burke G.; Bowman J.L.; Delaney D.A.	Geographic Drivers More Important Than Landscape Composition In Predicting Bee Beta Diversity And Community Structure	Ecosphere	10.1002/ecs2.4819
2024	Roque N.; Fernandez P.; Silveira C.; Vilas-Boas M.; Anjos O.	Using Analytic Hierarchy Process To Assess Beekeeping Suitability In Portuguese Controlled Areas: A First Approach	Insects	10.3390/insects15020091
2024	Demková L.; Hauptvogl M.; Oboňa J.; Bobuľská L.; Jančo I.; Harangozo Ľ.; Lakatošová J.; Kowalski S.; Árvay J.	Comprehensive Assessment Of Mercury Contamination In Bees, Bee Products And Moss And Lichen Bags	Ecotoxicology and Environmental Safety	10.1016/j.ecoenv.2024.117132
2024	Simões A.; Jesus T.A.D.; Dorini L.B.; Gomes-Jr L.	Generalization Method And System For Recommending Native Bee Species For Meliponiculture Practice	ACM International Conference Proceeding Series	10.1145/3658271.3658291
2024	Melin A.; Beale C.M.; Manning J.C.; Colville J.F.	Fine-Scale Bee Species Distribution Models: Hotspots Of Richness And Endemism In South Africa With Species-Area Comparisons	Insect Conservation and Diversity	10.1111/icad.12715
2024	Robinson J.L.	Project-Specific Bumble Bee Habitat Quality Assessment	MethodsX	10.1016/j.mex.2024.102571
2024	Schmidlová S.; Javůrková Z.; Tremlová B.; Hernik J.; Prus B.; Marcinčák S.; Marcinčáková D.; Štarha P.; Čížková H.; Kružík V.; Bodor Z.; Benedek C.; Titěra D.; Boržíková J.; Pospiech M.	Exploring The Influence Of Soil Types On The Mineral Profile Of Honey: Implications For Geographical Origin Prediction	Foods	10.3390/foods13132006
2024	Shahimoridi R.; Kazemi H.; Kamkar B.; Nadimi A.; Hosseinalizadeh M.; Yeganeh H.; Yildirim M.U.	Quantification And Mapping Of Regulating Ecosystem Services In Canola Agroecosystems (Case Study: Gorgan County, Iran)	Environmental Monitoring and Assessment	10.1007/s10661-024-13100-4
2024	Ruiz-Utrilla Z.P.; del-Val E.; Equihua J.; Cuervo-Robayo Á.P.	Risk Of Asian Hornet Invasion In Mexico: A Proposal For Invasive Species Risk Assessment From A Spatial Perspective	Biodiversity and Conservation	10.1007/s10531-024-02831-y
2024	Li K.; Fisher J.R.B.; Power A.G.; Iverson A.L.	A Map Of Pollinator Floral Resource Habitats In The Agricultural Landscape Of Central New York	One Ecosystem	10.3897/oneeco.9.e118634
2024	Shi J.; Wang X.; Chen Z.; Mao D.; Luo Y.	Spatial Distribution Of Two Acaricides And Five Neonicotinoids In Beehives And Surrounding Environments In China	Journal of Hazardous Materials	10.1016/j.jhazmat.2024.133892
2024	Mahdizadeh Gharakhanlou N.; Perez L.; Coallier N.	Mapping Crop Types For Beekeepers Using Sentinel-2 Satellite Image Time Series: Five Essential Crops In The Pollination Services	Remote Sensing	10.3390/rs16224225
2023	Richardson R.T.; Conflitti I.M.; Labuschagne R.S.; Hoover S.E.; Currie R.W.; Giovenazzo P.; Guarna M.M.; Pernal S.F.; Foster L.J.; Zayed A.	Land Use Changes Associated With Declining Honey Bee Health Across Temperate North America	Environmental Research Letters	10.1088/1748-9326/acd867

2023	Cotrina-Sanchez A.; García L.; Calle C.; Sari F.; Bandopadhyay S.; Rojas-Briceño N.B.; Meza-Mori G.; Torres Guzmán C.; Auquiñivín-Silva E.; Arellanos E.; Oliva M.	Multicriteria Analysis In Apiculture: A Sustainable Tool For Rural Development In Communities And Conservation Areas Of Northwest Peru	Land	10.3390/land12101900
2023	Moomen A.-W.; Odame-Appiah D.	Examining The Spatial Incongruity Between Mining Sector And Beekeeping Activities	Natural Resources Forum	10.1111/1477-8947.12274
2023	Prestby T.J.; Robinson A.C.; McLaughlin D.; Dudas P.M.; Grozinger C.M.	Characterizing User Needs For Beescape: A Spatial Decision Support Tool Focused On Pollinator Health	Journal of Environmental Management	10.1016/j.jenvman.2022.116416
2023	Signorelli D.; D'Auria L.J.; Di Stasio A.; Gallo A.; Siciliano A.; Esposito M.; De Felice A.; Rofrano G.	Application Of A Quality-Specific Environmental Risk Index For The Location Of Hives In Areas With Different Pollution Impacts	Agriculture (Switzerland)	10.3390/agriculture13050998
2023	Binney B.M.; Pragert H.; Foxwell J.; Gias E.; Birrell M.L.; Phiri B.J.; Quinn O.; Taylor M.; Ha H.J.; Hall R.J.	Genomic Analysis Of The Population Structure Of Paenibacillus Larvae In New Zealand	Frontiers in Microbiology	10.3389/fmicb.2023.1161926
2023	Fauzan R.; Wibawa J.C.; Muhammad Nursukma A.; Radliya N.R.	Web-Based Geographic Information System For Member Distribution Mapping And Honey Sales At Inspirator Lebah Madu Indonesia (Ilmi) With Prototype Methods	INCITEST 2023 - Proceedings of the 2023 International Conference on Informatics Engineering, Science and Technology	10.1109/INCITEST59455.2023.10396882
2023	Yaman Ş.; Yaman M.	Determination Of Suitable Beekeeping Places By Weighted Overlay Analysis: A Case Study Of Bolu, Türkiye; [Weighted Overlay Analizi Ile Uygun Arıcılık Yerlerinin Belirlenmesi: Bolu, Türkiye Örneği]	Uludag Aricilik Dergisi	10.31467/uluaricilik.1358531
2023	Tennakoon S.; Apan A.; Maraseni T.; Altarez R.D.D.	Decoding The Impacts Of Space And Time On Honey Bees: Gis Based Fuzzy Ahp And Fuzzy Overlay To Assess Land Suitability For Apiary Sites In Queensland, Australia	Applied Geography	10.1016/j.apgeog.2023.102951
2023	Abou-Shaara H.F.; Kelany M.M.	A Methodology To Assist In Locating Drone Congregation Area Using Remote Sensing Technique	Journal of Apicultural Research	10.1080/00218839.2021.1898786
2023	Sarvia F.; De Petris S.; Borgogno-Mondino E.	Mapping Melliferous Potential In Productive Honey Areas Through Spatial Tools: Towards A Rationalization Of Beekeeping	Ecological Informatics	10.1016/j.ecoinf.2023.102362
2023	Shaw J.; Cunningham C.; Harper S.; Ragazzon-Smith A.; Lythgoe P.R.; Walker T.R.	Biomonitoring Of Honey Metal(Loid) Pollution In Northwest England By Citizen Scientists	Environmental Advances	10.1016/j.envadv.2023.100406
2023	Chévez E.; Porter-Bolland L.; García-Frapolli E.; Landgrave R.; Revollo-Fernández D.	Bee Landscape Relations In Changing Contexts, Implications For Stingless Bee Management	Regional Environmental Change	10.1007/s10113-023-02093-4
2023	Quinlan G.M.; Miller D.A.W.; Grozinger C.M.	Examining Spatial And Temporal Drivers Of Pollinator Nutritional Resources: Evidence From Five Decades Of Honey Bee Colony Productivity Data	Environmental Research Letters	10.1088/1748-9326/acff0c
2023	Mercan Ç.	Land Suitability Assessment For Apiculture (Beekeeping) Activity Areas Using Geographic Information System And Ahp: A Case Study Bitlis/Türkiye; [Coğrafi Bilgi Sistemi Ve Ahp Ile Aricilik Faaliyet Alanlari Için Arazi Uygunluk Değerlendirmesi: Bitlis/Türkiye Örneği]	Uludag Aricilik Dergisi	10.31467/uluaricilik.1245078
2023	Kotovs D.; Zacepins A.	Gis-Based Interactive Map To Improve Scheduling Beekeeping Activities	Agriculture (Switzerland)	10.3390/agriculture13030669
2022	Amro A.M.; Abou-Shaara H.F.	Using Geographical Information Systems And Remote Sensing To Detect Resin-Rich Areas For Propolis Production From Apiaries; [An Kovanlanndan Propolis Üretimi Için Reçine Bakimmdan Zengin Alanlan Tespit Etmek Için Coğrafi Bilgi Sistemlerini Ve Uzaktan Algilamayi Kullanma]	Uludag Aricilik Dergisi	10.31467/uluaricilik.1027232
2022	Insolia L.; Molinari R.; Rogers S.R.; Williams G.R.; Chiaromonte F.; Calovi M.	Honey Bee Colony Loss Linked To Parasites, Pesticides And Extreme Weather Across The United States	Scientific Reports	10.1038/s41598-022-24946-4

2022	Overturf K.A.; Steinhauer N.; Molinari R.; Wilson M.E.; Watt A.C.; Cross R.M.; vanEngelsdorp D.; Williams G.R.; Rogers S.R.	Winter Weather Predicts Honey Bee Colony Loss At The National Scale	Ecological Indicators	10.1016/j.ecolind.2022.109709
2022	Abou-Shaara H.F.; Amiri E.; Parys K.A.	Tracking The Effects Of Climate Change On The Distribution Of Plecia Nearctica (Diptera, Bibionidae) In The Usa Using Maxent And Gis	Diversity	10.3390/d14080690
2022	Quinlan G.M.; Sponsler D.; Gaines-Day H.R.; McMinn-Sauder H.B.G.; Otto C.R.V.; Smart A.H.; Colin T.; Gratton C.; Isaacs R.; Johnson R.; Milbrath M.O.; Grozinger C.M.	Grassy-Herbaceous Land Moderates Regional Climate Effects On Honey Bee Colonies In The Northcentral Us	Environmental Research Letters	10.1088/1748-9326/ac7063
2022	Douglas M.R.; Baisley P.; Soba S.; Kammerer M.; Lonsdorf E.V.; Grozinger C.M.	Putting Pesticides On The Map For Pollinator Research And Conservation	Scientific Data	10.1038/s41597-022-01584-z
2022	Kougioumoutzis K.; Kaloveloni A.; Petanidou T.	Assessing Climate Change Impacts On Island Bees: The Aegean Archipelago	Biology	10.3390/biology11040552
2022	Blaydes H.; Gardner E.; Whyatt J.D.; Potts S.G.; Armstrong A.	Solar Park Management And Design To Boost Bumble Bee Populations	Environmental Research Letters	10.1088/1748-9326/ac5840
2022	Alkhalaf A.A.	Utilizing Ecological Modeling To Follow The Potential Spread Of Honey Bee Pest (Megaselia Scalaris) From Nearby Countries Towards Saudi Arabia Under Climate Change Conditions	Diversity	10.3390/d14040261
2022	Dabove P.; Di Pietra V.	The Use Of Open Source Software For Monitoring Bee Diversity In Natural Systems: The Beems Project	International Archives of the Photogrammetry, Remote Sensing and Spatial Information Sciences - ISPRS Archives	10.5194/isprs-archives-XLVIII-4-W1-2022-111-2022
2022	Abou-Shaara H.F.; Al-Khalaf A.A.	Using Maximum Entropy Algorithm To Analyze Current And Future Distribution Of The Asian Hornet, Vespa Velutina, In Europe And North Africa Under Climate Change Conditions	Journal of the Entomological Research Society	10.51963/jers.v24i1.2011
2022	Elmastas N; Ölmez I; Vural E	Suitability Analysis Of Apiculture (Beekeeping) Activity Areas With Multi-Criteria Method: A Case Study Of Adıyaman	JOURNAL OF GEOGRAPHY-COGRAFYA DERGISI	10.26650/JGEOG2022-894419
2022	Rubtsova TA; Zubareva AM	Characteristics Of Melliferous Lands And Their Fire Hazard In The Jewish Autonomous Region	Lesnoy Zhurnal = Russian Forestry Journal	10.37482/0536-1036-2022-3-32-43
2021	Komasilova O.; Komasilovs V.; Kviesis A.; Zacepins A.	Model For Finding The Number Of Honey Bee Colonies Needed For The Optimal Foraging Process In A Specific Geographical Location	PeerJ	10.7717/peerj.12178
2021	Al-Khalaf A.A.	Modeling The Potential Distribution Of The Predator Of Honey Bees, Palarus Latifrons, In The Arabian Deserts Using Maxent And Gis	Saudi Journal of Biological Sciences	10.1016/j.sjbs.2021.06.012
2021	Joshi M.Y.; Rivière L.; Mahy G.; Teller J.	Effectiveness Of Green Roofs In Strengthening Ecological Network	International Archives of the Photogrammetry, Remote Sensing and Spatial Information Sciences - ISPRS Archives	10.5194/isprs-archives-XLVI-4-W1-2021-51-2021
2021	Pless R.; Ferreira S.; Bergstrom J.; Rabinowitz A.N.	Spatial And Temporal Trends In The Economic Value Of Biotic Pollination Services In Georgia, Usa: 2009-2017	Journal of Agricultural and Applied Economics	10.1017/aae.2021.11
2021	Olynyk M.; Westwood A.R.; Koper N.	Effects Of Natural Habitat Loss And Edge Effects On Wild Bees And Pollination Services In Remnant Prairies	Environmental Entomology	10.1093/ee/nvaa186
2021	Jamal Z.A.; Abou-Shaara H.F.; Qamer S.; Alhumaidi Alotaibi M.; Ali Khan K.; Fiaz Khan M.;	Future Expansion Of Small Hive Beetles, Aethina Tumida, Towards North Africa And South Europe Based On Temperature Factors Using Maximum Entropy Algorithm	Journal of King Saud University - Science	10.1016/j.jksus.2020.101242

	Amjad Bashir M.; Hannan A.; AL-Kahtani S.N.; Taha E.-K.A.; Ishtiaq Anjum S.; Attaullah M.; Raza G.; Ansari M.J.			
2021	Ochungo P.; Veldtman R.; Abdel-Rahman E.M.; Raina S.; Muli E.; Landmann T.	Multi-Sensor Mapping Of Honey Bee Habitats And Fragmentation In Agro-Ecological Landscapes In Eastern Kenya	Geocarto International	10.1080/10106049.2019.1629645
2021	Hatfield R.G.; Strange J.P.; Koch J.B.; Jepsen S.; Stapleton I.	Neonicotinoid Pesticides Cause Mass Fatalities Of Native Bumble Bees: A Case Study From Wilsonville, Oregon, United States	Environmental Entomology	10.1093/ee/nvab059
2021	Calovi M.; Grozinger C.M.; Miller D.A.; Goslee S.C.	Summer Weather Conditions Influence Winter Survival Of Honey Bees (Apis Mellifera) In The Northeastern United States	Scientific Reports	10.1038/s41598-021-81051-8
2021	Dorey J.B.; Rebola C.M.; Davies O.K.; Prendergast K.S.; Parslow B.A.; Hogendoorn K.; Leijs R.; Hearn L.R.; Leitch E.J.; O'Reilly R.L.; Marsh J.; Woinarski J.C.Z.; Caddy-Retalic S.	Continental Risk Assessment For Understudied Taxa Post-Catastrophic Wildfire Indicates Severe Impacts On The Australian Bee Fauna	Global Change Biology	10.1111/gcb.15879
2021	Kurmanov R.G.; Galeev R.I.	Mapping Honeylands Of The European Part Of Russia; [Картографирование Медоносных Угодий Европейской Части России]	Vestnik Moskovskogo Universiteta, Seriya Geografiya	
2021	Dorey J.B.	Missing For Almost 100 Years: The Rare And Potentially Threatened Bee, Pharohylaeus Lactiferus (Hymenoptera, Colletidae)	Journal of Hymenoptera Research	10.3897/JHR.81.59365
2021	Marnasidis S.; Kantartzis A.; Malesios C.; Hatjina F.; Arabatzis G.; Verikouki E.	Mapping Priority Areas For Apiculture Development With The Use Of Geographical Information Systems	Agriculture (Switzerland)	10.3390/agriculture11020182
2021	Mutinelli F.; Mazzucato M.; Barbujani M.; Carpana E.; Di Salvo V.; Gardi T.; Greco D.; Bonizzoni L.; Benvenuti M.; Casarotto C.; Bortolotti L.; Costa C.	The Italian National Beekeeping Registry (Bdna) As A Tool To Identify Areas Suitable For Controlled Mating Of Honey Bees In Italy	Applied Sciences (Switzerland)	10.3390/app11115279
2021	Meier V.S.; Walz U.; Syrbe R.-U.; Grunewald K.	Indicators For Pollination Potential- The Nationwide Habitat Potential For Wild Bees; [Das Bundesweite Habitatpotenzial Für Wildbienen Ein Indikator Für Die Bestäubungsleistung]	Naturschutz und Landschaftsplanung	10.1399/NuL.2021.06.01
2021	Khanna V.; Jordan A.; Patch H.M.; Grozinger C.M.	Economic Dependence And Vulnerability Of United States Agricultural Sector On Insect-Mediated Pollination Service	Environmental Science and Technology	10.1021/acs.est.0c04786
2021	Robinson A.C.; Peeler J.L.; Prestby T.; Goslee S.C.; Anton K.; Grozinger C.M.	Beescape: Characterizing User Needs For Environmental Decision Support In Beekeeping	Ecological Informatics	10.1016/j.ecoinf.2021.101366
2021	Brodschneider R.; Kalcher-Sommersguter E.; Kuchling S.; Dietemann V.; Gray A.; Božič J.; Briedis A.; Carreck N.L.; Chlebo R.; Crailsheim K.; Coffey M.F.; Dahle B.; González-Porto A.V.; Filipi J.; de Graaf D.C.; Hatjina F.; Ioannidis P.; Ion N.; Jørgensen A.S.; Kristiansen P.; Lecocq A.; Odoux J.-F.; Özkirim A.; Peterson M.; Podrižnik B.; Rašić S.; Retschnig G.; Schiesser A.; Tosi S.; Vejsnæs F.; Williams G.; van der Steen J.J.M.	Csi Pollen: Diversity Of Honey Bee Collected Pollen Studied By Citizen Scientists	Insects	10.3390/insects12110987
2021	Kumar P.B.; Prasad T.K.	Application Of Multi-Criteria Decision Analysis (Mcda) To Apiculture Potential Assessment: A Case Study Of Thiruvananthapuram Corporation, Kerala, India	Transactions of the Institute of Indian Geographers	
2021	Cappa V.; Cerioli M.P.; Scaburri A.; Tironi M.; Farioli M.; Nassuato C.; Bellini S.	Analysis Of Bee Population Decline In Lombardy During The Period 2014–2016 And Identification Of High-Risk Areas	Pathogens	10.3390/pathogens10081004

2021	Abou-Shaara H.F.	Gis Analysis To Locate More Suitable Wintering Areas For Honey Bee Colonies In Agricultural And Desert Lands	African Entomology	10.4001/003.029.0405
2021	Cakir G	Determination Of The Honey Production Areas Within Land Use Types: A Case Study Tercan, Turkey	FRESENIUS ENVIRONMENTAL BULLETIN	
2020	Komasilova O.; Komasilovs V.; Kviesis A.; Bumanis N.; Mellmann H.; Zacepins A.	Model For The Bee Apiary Location Evaluation	Agronomy Research	10.15159/AR.20.090
2020	Smith K.E.; Weis D.	Evaluating Spatiotemporal Resolution Of Trace Element Concentrations And Pb Isotopic Compositions Of Honeybees And Hive Products As Biomonitors For Urban Metal Distribution	GeoHealth	10.1029/2020GH000264
2020	Akinduti P.A.; Ejilude O.; Olugbuyiro J.; Adewale A.G.; Onagbesan O.; Afolabi O.	Geospatial Investigation Of Nigerian Honey And Detection Of Anti-Enteric Biomarker	Evidence-based Complementary and Alternative Medicine	10.1155/2020/9817673
2020	Douglas M.R.; Sponsler D.B.; Lonsdorf E.V.; Grozinger C.M.	County-Level Analysis Reveals A Rapidly Shifting Landscape Of Insecticide Hazard To Honey Bees (Apis Mellifera) On Us Farmland	Scientific Reports	10.1038/s41598-019-57225-w
2020	Cohen H.; McFrederick Q.S.; Philpott S.M.	Environment Shapes The Microbiome Of The Blue Orchard Bee, Osmia Lignaria : Rrh: Environmental Drivers Of Bee Microbiome	Microbial Ecology	10.1007/s00248-020-01549-y
2020	Birdshire K.R.; Carper A.L.; Briles C.E.	Bee Community Response To Local And Landscape Factors Along An Urban-Rural Gradient	Urban Ecosystems	10.1007/s11252-020-00956-w
2020	Dorey B.; Fagan-Jeffries E.P.; Stevens M.I.; Schwarz M.P.	Morphometric Comparisons And Novel Observations Of Diurnal And Low-Light-Foraging Bees	Journal of Hymenoptera Research	10.3897/JHR.79.57308
2020	Sari F.	Assessment Of Land - Use Change Effects On Future Beekeeping Suitability Via Ca-Markov Prediction Model	Journal of Apicultural Science	10.2478/jas-2020-0020
2020	Barker B.	Utilities Abuzz	EPRI Journal	
2020	Sarı F.; Ceylan D.A.; Özcan M.M.; Özcan M.M.	A Comparison Of Multicriteria Decision Analysis Techniques For Determining Beekeeping Suitability	Apidologie	10.1007/s13592-020-00736-7
2020	Sari F.; Kandemir I.; Ceylan D.A.	Integration Of Ndvi Imagery And Crop Coverage Registration System For Apiary Schedule	Journal of Apicultural Science	10.2478/jas-2020-0011
2020	Van Esch L.; De Kok J.-L.; Janssen L.; Buelens B.; De Smet L.; de Graaf D.C.; Engelen G.	Multivariate Landscape Analysis Of Honey Bee Winter Mortality In Wallonia, Belgium	Environmental Modeling and Assessment	10.1007/s10666-019-09682-w
2020	Dufour C.; Fournier V.; Giovenazzo P.	The Impact Of Lowbush Blueberry (Vaccinium Angustifolium Ait.) And Cranberry (Vaccinium Macrocarpon Ait.) Pollination On Honey Bee (Apis Mellifera L.) Colony Health Status	PLoS ONE	10.1371/journal.pone.0227970
2020	Sari F.; Kandemir İ.; Ceylan D.A.; Gül A.	Using Ahp And Promethee Multi-Criteria Decision Making Methods To Define Suitable Apiary Locations	Journal of Apicultural Research	10.1080/00218839.2020.1718341
2020	Salehizadeh A.; Khodagholi M.; Gandomkar A.	Temperature Conditions For Determination Of Beekeeping Regions In The Light Of Climate Change. Case Study: Fars Province	Environmental and Climate Technologies	10.2478/rtuect-2020-0006
2020	Peña M.A.; Zegers D.A.	Assessment Of Vegetation Aptitude To Organic Beekeeping; [Valoración De La Aptitud Vegetal Para La Apicultura Orgánica]	Revista Geografica de Chile Terra Australis	10.23854/07199562.2020561.Pena129
2020	Sari F.; Sari F.K.	Land Use Change Assesment For Beekeeping In Southeast Anatolia; [Aricilik Icin Arazi Kullanim Deäa&lâikliklerinin Guneydoäa&U Anadolu Ällerinde Äncelenmesi]	Uludag Aricilik Dergisi	10.31467/ULUARICILIK.684608
2019	Abou-Shaara H.F.; Eid K.S.A.	Increasing The Profitability Of Propolis Production In Honey Bee Colonies By Utilizing Remote Sensing Techniques To Spot Locations Of Trees As Potential Sources Of Resin	Remote Sensing Letters	10.1080/2150704X.2019.1633488

2019	Von Büren R.S.; Oehen B.; Kuhn N.J.; Erler S.	High-Resolution Maps Of Swiss Apiaries And Their Applicability To Study Spatial Distribution Of Bacterial Honey Bee Brood Diseases	PeerJ	10.7717/peerj.6393
2019	Kupsch S.; Ventayen R.J.M.; Dela Cruz J.S.	Plotting A Healthy Apiary Location Using Webgis	ACM International Conference Proceeding Series	10.1145/3323716.3323726
2019	Salatnaya H.; Widiatmaka; Sumantri C.; Kahoho S.; Fuah A.M.	Potential Growth Of Meliponiculture In West Halmahera, Indonesia	IOP Conference Series: Earth and Environmental Science	10.1088/1755-1315/399/1/012046
2019	Calatayud-Vernich P.; Calatayud F.; Simó E.; Pascual Aguilar J.A.; Picó Y.	A Two-Year Monitoring Of Pesticide Hazard In-Hive: High Honey Bee Mortality Rates During Insecticide Poisoning Episodes In Apiaries Located Near Agricultural Settings	Chemosphere	10.1016/j.chemosphere.2019.05.170
2019	Awad A.M.; Owayss A.A.; Iqbal J.; Raweh H.S.A.; Alqarni A.S.	Gis Approach For Determining The Optimum Spatiotemporal Plan For Beekeeping And Honey Production In Hot-Arid Subtropical Ecosystems	Journal of Economic Entomology	10.1093/jee/toz002
2019	Arany I.; VARI A.; Aszalos R.; Kelemen K.; Kelemen M.A.; Bone G.; Lellei-Kovacz E.; Czusz B.	Diversity Of Flower-Rich Habitats As A Persistent Source Of Healthy Diet For Honey Bees	European Journal of Geography	
2019	Campana C.; Peralta C.; Cecconello J.C.; Pons D.H.; Uranga J.; Scavuzzo M.C.; Ferral A.	Geospatial Tools Applied To The Generation Of An Aptitude Map For The Development Of Beekeeping Activity In San Javier, Córdoba, Argentina	2019 18th Workshop on Information Processing and Control, RPIC 2019	10.1109/RPIC.2019.8882180
2019	Espíndola A.; Pliscoff P.	The Relationship Between Pollinator Visits And Climatic Suitabilities In Specialized Pollination Interactions	Annals of the Entomological Society of America	10.1093/aesa/say042
2018	Samuelson A.E.; Leadbeater E.	A Land Classification Protocol For Pollinator Ecology Research: An Urbanization Case Study	Ecology and Evolution	10.1002/ece3.4087
2018	Berg C.J.; Peter King H.; Delenstarr G.; Kumar R.; Rubio F.; Glaze T.	Glyphosate Residue Concentrations In Honey Attributed Through Geospatial Analysis To Proximity Of Large-Scale Agriculture And Transfer Off-Site By Bees	PLoS ONE	10.1371/journal.pone.0198876
2018	Burr A.; Schaeg N.; Hall D.M.	Assessing Residential Front Yards Using Google Street View And Geospatial Video: A Virtual Survey Approach For Urban Pollinator Conservation	Applied Geography	10.1016/j.apgeog.2018.01.010
2018	Daher-Hjaij N.Y.; Al-Mohamed R.; Al-Ghamaz F.; Al-Zoubi M.M.; Qrebsa M.S.	Distribution Of Honey And Pollen Forestry Trees Important To Honey Bee Along The Syrian Coast Using Geographic Information Systems	Arab Journal of Plant Protection	10.22268/AJPP-036.1.014020
2018	Ausseil A.-G.E.; Dymond J.R.; Newstrom L.	Mapping Floral Resources For Honey Bees In New Zealand At The Catchment Scale	Ecological Applications	10.1002/eap.1717
2018	Drummond F.A.; Ballman E.S.; Eitzer B.D.; Clos B.D.; Dill J.	Exposure Of Honey Bee (Apis Mellifera L.) Colonies To Pesticides In Pollen, A Statewide Assessment In Maine	Environmental Entomology	10.1093/ee/nvy023
2018	Leonard R.J.; Wat K.K.Y.; McArthur C.; Hochuli D.F.	Urbanisation And Wing Asymmetry In The Western Honey Bee (Apis Mellifera, Linnaeus 1758) At Multiple Scales	PeerJ	10.7717/peerj.5940
2018	Graham K.K.; MacLean M.G.	Presence-Only Modeling Is Ill-Suited For A Recent Generalist Invader, Anthidium Manicatum	Ecological Indicators	10.1016/j.ecolind.2018.02.002
2017	Glaum P.; Simao M.-C.; Vaidya C.; Fitch G.; Iulinao B.	Big City Bombus: Using Natural History And Land-Use History To Find Significant Environmental Drivers In Bumble-Bee Declines In Urban Development	Royal Society Open Science	10.1098/rsos.170156
2017	Switanek M.; Crailsheim K.; Truhetz H.; Brodschneider R.	Modelling Seasonal Effects Of Temperature And Precipitation On Honey Bee Winter Mortality In A Temperate Climate	Science of the Total Environment	10.1016/j.scitotenv.2016.11.178
2017	McKechnie I.M.; Thomsen C.J.M.; Sargent R.D.	Forested Field Edges Support A Greater Diversity Of Wild Pollinators In Lowbush Blueberry (Vaccinium Angustifolium)	Agriculture, Ecosystems and Environment	10.1016/j.agee.2016.12.005
2017	Nguyen H.; Ketchell S.; Engelke U.; Thomas B.; De Souza P.	Holobee: Augmented Reality Based Bee Drift Analysis	Adjunct Proceedings of the 2017 IEEE International Symposium on Mixed and Augmented Reality, ISMAR-Adjunct 2017	10.1109/ISMAR-Adjunct.2017.38

2017	Pantoja G.; Gómez M.; Contreras C.; Grimau L.; Montenegro G.	Determination Of Suitable Zones For Apitourism Using Multi-Criteria Evaluation In Geographic Information Systems: A Case Study In The O'Higgins Region, Chile	Ciencia e Investigacion Agraria	10.7764/rcia.v44i2.1712
2017	Adgaba N.; Alghamdi A.; Sammoud R.; Shenkute A.; Tadesse Y.; Ansari M.J.; Sharma D.; Hepburn C.	Determining Spatio-Temporal Distribution Of Bee Forage Species Of Al-Baha Region Based On Ground Inventorying Supported With Gis Applications And Remote Sensed Satellite Image Analysis	Saudi Journal of Biological Sciences	10.1016/j.sjbs.2017.01.009
2017	Zoccali P.; Malacrinò A.; Campolo O.; Laudani F.; Algeri G.M.; Giunti G.; Strano C.P.; Benelli G.; Palmeri V.	A Novel Gis-Based Approach To Assess Beekeeping Suitability Of Mediterranean Lands	Saudi Journal of Biological Sciences	10.1016/j.sjbs.2017.01.062
2017	Sponsler D.B.; Matcham E.G.; Lin C.-H.; Lanterman J.L.; Johnson R.M.	Spatial And Taxonomic Patterns Of Honey Bee Foraging: A Choice Test Between Urban And Agricultural Landscapes	Journal of Urban Ecology	10.1093/jue/juw008
2017	Keeling M.J.; Franklin D.N.; Datta S.; Brown M.A.; Budge G.E.	Predicting The Spread Of The Asian Hornet (Vespa Velutina) Following Its Incursion Into Great Britain	Scientific Reports	10.1038/s41598-017-06212-0
2017	Sarl F.; Ceylan D.A.	Site Suitability Analysis For Beekeeping Via Analythical Hyrearchy Process, Konya Example	ISPRS Annals of the Photogrammetry, Remote Sensing and Spatial Information Sciences	10.5194/isprs-annals-IV-4-W4-345-2017
2017	Castellanos-Potenciano B.; Gallardo-López F.; Díaz-Padilla G.; Pérez-Vázquez A.; Landeros-Sánchez C.	Spatio-Temporal Mobility Of Apiculture Affected By The Climate Change In The Beekeeping Of The Gulf Of Mexico	Applied Ecology and Environmental Research	10.15666/aeer/1504_163175
2017	Widiatmaka; Ambarwulan Wiwin; Sjamsudin Chandrasa E; Syaufina Lailan	Geographic Information System And Analytical Hierarchy Process For Land Use Planning Of Beekeeping In Forest Margin Of Bogor Regency, Indonesia	Jurnal Silvikultur Tropika	
2016	Heimbach F.; Russ A.; Schimmer M.; Born K.	Large-Scale Monitoring Of Effects Of Clothianidin Dressed Oilseed Rape Seeds On Pollinating Insects In Northern Germany: Implementation Of The Monitoring Project And Its Representativeness	Ecotoxicology	10.1007/s10646-016-1724-9
2016	Schochet A.B.; Hung K.-L.J.; Holway D.A.	Bumble Bee Species Exhibit Divergent Responses To Urbanisation In A Southern California Landscape	Ecological Entomology	10.1111/een.12343
2016	Cusser S.; Neff J.L.; Jha S.	Natural Land Cover Drives Pollinator Abundance And Richness, Leading To Reductions In Pollen Limitation In Cotton Agroecosystems	Agriculture, Ecosystems and Environment	10.1016/j.agee.2016.04.020
2016	Abou-Shaara HF	Expectations About The Potential Impacts Of Climate Change On Honey Bee Colonies In Egypt	Journal of Apiculture	
2016	Kaur Parvinder; Mishra Atul A; Lal Deepak	Honey Characterization Based On Physicochemical Parameters Using Gis Techniques: A Case Study In Selected States Of Northern India	Journal of Food Processing & Technology	10.4172/2157-7110.1000626
2016	Triantomo Varian; Widiatmaka; Fuah Asnath M	Land Use Planning For Beekeeping Using Geographic Information System In Sukabumi Regency, West Java	Jurnal Pengelolaan Sumberdaya Alam dan Lingkungan	10.29244/jpsl.6.2.168
2016	Yari R; Heshmati GH; Rafiei H	Assessing The Potential Of Beekeeping And Determination Of Attractiveness Range Plants Used Bee By Using Geographic Information System In Char-Bagh Summer Rangelands, Golestan	RS & GIS for Natural Resources	
2016	Fernandez P; Roque N; Anjos O	Spatial Multicriteria Decision Analysis To Potential Beekeeping Assessment Case Study Montesinho Natural Park Portugal	19th AGILE Conference on Geographic Information Science	
2015	Barfield A.S.; Bergstrom J.C.; Ferreira S.; Covich A.P.; Delaplane K.S.	An Economic Valuation Of Biotic Pollination Services In Georgia	Journal of Economic Entomology	10.1093/jee/tou045

2015	Koch J.B.; Lozier J.; Strange J.P.; Ikerd H.; Griswold T.; Cordes N.; Solter L.; Stewart I.; Cameron S.A.	Usbombus, A Database Of Contemporary Survey Data For North American Bumble Bees (Hymenoptera, Apidae, Bombus) Distributed In The United States	Biodiversity Data Journal	10.3897/BDJ.3.e6833
2015	Galindo-Cardona A.; Monmany A.C.; Diaz G.; Giray T.	A Landscape Analysis To Understand Orientation Of Honey Bee (Hymenoptera: Apidae) Drones In Puerto Rico	Environmental Entomology	10.1093/ee/nvv099
2015	Sponsler D.B.; Johnson R.M.	Honey Bee Success Predicted By Landscape Composition In Ohio, Usa	PeerJ	10.7717/peerj.838
2015	Abou-Shaara HF	Suitability Of Current And Future Conditions To Apiculture In Egypt Using Geographical Information System	Journal of Agricultural Informatics	10.17700/jai.2015.6.2.189
2014	Camargo S.C.; Garcia R.C.; Feiden A.; De Vasconcelos E.S.; Pires B.G.; Hartleben A.M.; De Moraes F.J.; De Oliveira L.; Giasson J.; Mittanck E.S.; Gremaschi J.R.; Pereira D.J.	Implementation Of A Geographic Information System (Gis) For The Planning Of Beekeeping In The West Region Of Paraná	Anais da Academia Brasileira de Ciencias	10.1590/0001-3765201420130278
2014	Resende H.C.; Fernandes-Salomão T.M.; Tavares M.G.; Campos L.	Geographic Distribution, Key Challenges, And Prospects For The Conservation Of Threatened Stingless Bee Melipona Capixaba Moure And Camargo (Hymenoptera: Apidae: Meliponini)	Sociobiology	10.13102/sociobiology.v61i4.529-535
2014	Bendini J.N.; Orsi R.O.; Bendini H.N.; Souza D.C.; e Salum R.B.	Spatial Characterization Of Beekeeping In The Micro Region Of Campos Do Jordao, São Paulo, Brazil; [Caracterização Espacial Da Atividade Apícola Na Microrregião De Campos Do Jordão, São Paulo, Brasil]	Archivos de Zootecnia	10.4321/s0004-05922014000400012
2014	Griswold T.; Gonzalez V.H.; Ikerd H.	Anthwest, Occurrence Records For Wool Carder Bees Of The Genus Anthidium (Hymenoptera, Megachilidae, Anthidiini) In The Western Hemisphere	ZooKeys	10.3897/zookeys.408.5633
2014	Gordon R.; Bresolin-Schott N.; East I.J.	Nomadic Beekeeper Movements Create The Potential For Widespread Disease In The Honeybee Industry	Australian Veterinary Journal	10.1111/avj.12198
2014	Piroux M.; Lambert O.; Puyo S.; Farrera I.; Thorin C.; L'Hostis M.; Vigues B.; Bastian S.	Correlating The Pollens Gathered By Apis Mellifera With The Landscape Features In Western France	Applied Ecology and Environmental Research	10.15666/aeer/1202_423439
2014	Radović D.I.; Lazarević K.B.; Trifković J.D.; Andrić F.L.; Tešić Ž.L.; Andelković I.B.; Nedić N.M.; Stanimirović Z.; Stevanović J.; Ćurčić B.P.M.; Milojković-Opsenica D.M.	Gis Techology In Regional Recognition Of The Distribution Pattern Of Multifloral Honey: The Chemical Traits In Serbia	Archives of Biological Sciences	10.2298/ABS1402935R
2014	Brown J.C.; De Oliveira M.L.	The Impact Of Agricultural Colonization And Deforestation On Stingless Bee (Apidae: Meliponini) Composition And Richness In Rondônia, Brazil	Apidologie	10.1007/s13592-013-0236-3
2014	Saifuddin M.; Jha S.	Colony-Level Variation In Pollen Collection And Foraging Preferences Among Wild-Caught Bumble Bees (Hymenoptera: Apidae)	Environmental Entomology	10.1603/EN13261
2013	Spleen A.M.; Lengerich E.J.; Rennich K.; Caron D.; Rose R.; Pettis J.S.; Henson M.; Wilkes J.T.; Wilson M.; Stitzinger J.; Lee K.; Andree M.; Snyder R.; Van Engelsdorp D.	A National Survey Of Managed Honey Bee 2011-12 Winter Colony Losses In The United States: Results From The Bee Informed Partnership	Journal of Apicultural Research	10.3896/IBRA.1.52.2.07
2013	Barbet-Massin M.; Rome Q.; Muller F.; Perrard A.; Villemant C.; Jiguet F.	Climate Change Increases The Risk Of Invasion By The Yellow-Legged Hornet	Biological Conservation	10.1016/j.biocon.2012.09.015
2013	Cristina Giannini T.; Acosta A.L.; Silva C.I.D.; de Oliveira P.E.A.M.; Imperatriz-Fonseca V.L.; Saraiva A.M.	Identifying The Areas To Preserve Passion Fruit Pollination Service In Brazilian Tropical Savannas Under Climate Change	Agriculture, Ecosystems and Environment	10.1016/j.agee.2013.03.003

2013	Schürch R.; Couvillon M.J.; Burns D.D.R.; Tasman K.; Waxman D.; Ratnieks F.L.W.	Incorporating Variability In Honey Bee Waggle Dance Decoding Improves The Mapping Of Communicated Resource Locations	Journal of Comparative Physiology A: Neuroethology, Sensory, Neural, and Behavioral Physiology	10.1007/s00359-013-0860-4
2013	Abou-Shaara H.F.; Al-Ghamdi A.A.; Mohamed A.A.	A Suitability Map For Keeping Honey Bees Under Harsh Environmental Conditions Using Geographical Information System	World Applied Sciences Journal	10.5829/idosi.wasj.2013.22.08.7384
2013	Ferrauto G.; Costa R.M.S.; Pavone P.; Cantarella G.L.	Human Impact Assessment On The Sicilian Agroecosystems Through The Evaluation Of Melliferous Areas	Annali di Botanica	10.4462/annbotrm-10305
2013	Lazarević K.B.; Trifković J.D.; Andrić F.Lj.; Tešić Ž.L.; Andelković I.B.; Radović D.I.; Nedić N.M.; Milojković-Opsenica D.M.	Quality Parameters And Pattern Recognition Methods As A Tool In Tracing The Regional Origin Of Multifloral Honey	Journal of the Serbian Chemical Society	10.2298/JSC130701099L
2013	Abou-Shaara HF	Wintering Map For Honey Bee Colonies In El-Behera Governorate, Egypt By Using Geographical Information System (Gis)	Journal of Applied Sciences and Environmental Management	10.4314/jasem.v17i3.9
2013	Abou-Shaara HF	A Morphometry Map And A New Method For Honey Bee Morphometric Analysis By Using The Arcgis	Arthropods	
2012	Giannini T.C.; Acosta A.L.; Garófalo C.A.; Saraiva A.M.; Alves-dos-Santos I.; Imperatriz-Fonseca V.L.	Pollination Services At Risk: Bee Habitats Will Decrease Owing To Climate Change In Brazil	Ecological Modelling	10.1016/j.ecolmodel.2012.06.035
2012	Delgado D.L.; Pérez M.E.; Galindo-Cardona A.; Giray T.; Restrepo C.	Forecasting The Influence Of Climate Change On Agroecosystem Services: Potential Impacts On Honey Yields In A Small-Island Developing State	Psyche (London)	10.1155/2012/951215
2012	Ibrahim I.F.; Balasundram S.K.; Abdullah N.-A.P.; Sood A.M.; Mardan M.; Saberioon M.M.	The Spatial Distribution Of Apis Dorsata Host Plants Using An Integrated Geographical Information System-Remote Sensing Approach	American Journal of Agricultural and Biological Science	10.3844/ajabssp.2012.396.406
2012	Halbich Č.; Vostrovský V.	Monitoring Of Infection Pressure Of American Foulbrood Disease By Means Of Google Maps	Agris On-line Papers in Economics and Informatics	
2012	Williams N.M.; Regetz J.; Kremen C.	Landscape-Scale Resources Promote Colony Growth But Not Reproductive Performance Of Bumble Bees	Ecology	10.1890/11-1006.1
2012	Henry M.; Fröchen M.; Maillet-Mezeray J.; Breyne E.; Allier F.; Odoux J.-F.; Decourtye A.	Spatial Autocorrelation In Honeybee Foraging Activity Reveals Optimal Focus Scale For Predicting Agro-Environmental Scheme Efficiency	Ecological Modelling	10.1016/j.ecolmodel.2011.11.015
2012	Tipple B.J.; Chesson L.A.; Erkkila B.R.; Cerling T.E.; Ehleringer J.R.	B-Hive: Beeswax Hydrogen Isotopes As Validation Of Environment, Part Ii. Compound-Specific Hydrogen Isotope Analysis	Food Chemistry	10.1016/j.foodchem.2012.02.106
2012	Vanengelsdorp D.; Caron D.; Hayes J.; Underwood R.; Henson M.; Rennich K.; Spleen A.; Andree M.; Snyder R.; Lee K.; Roccasecca K.; Wilson M.; Wilkes J.; Lengerich E.; Pettis J.	A National Survey Of Managed Honey Bee 2010-11 Winter Colony Losses In The Usa: Results From The Bee Informed Partnership	Journal of Apicultural Research	10.3896/IBRA.1.51.1.14
2012	Choi M.-B.; Kim J.-K.; Lee J.-W.	Increase Trend Of Social Hymenoptera (Wasps And Honeybees) In Urban Areas, Inferred From Moving-Out Case By 119 Rescue Services In Seoul Of South Korea	Entomological Research	10.1111/j.1748-5967.2012.00472.x
2012	Amiri F; Shariff ABM	Application Of Geographic Information Systems In Landuse Suitability Evaluation For Beekeeping: A Case Study Of Vahregan Watershed (Iran)	African Journal of Agricultural Research	10.5897/AJAR10.1037
2011	Stohlgren T.J.; Jarnevich C.S.; Esaias W.E.; Morisette J.T.	Bounding Species Distribution Models	Current Zoology	10.1093/czoolo/57.5.642
2011	Watson J.C.; Wolf A.T.; Ascher J.S.	Forested Landscapes Promote Richness And Abundance Of Native Bees (Hymenoptera: Apoidea: Anthophila) In Wisconsin Apple Orchards	Environmental Entomology	10.1603/EN10231
2011	Amiri F.; Mohamed Shariff A.R.B.; Arekhi S.	An Approach For Rangeland Suitability Analysis To Apiculture Planning In Gharah Aghach Region, Isfahan-Iran	World Applied Sciences Journal	

2011	Villemant C.; Barbet-Massin M.; Perrard A.; Muller F.; Gargominy O.; Jiguet F.; Rome Q.	Predicting The Invasion Risk By The Alien Bee-Hawking Yellow-Legged Hornet Vespa Velutina Nigrithorax Across Europe And Other Continents With Niche Models	Biological Conservation	10.1016/j.biocon.2011.04.009
2011	Everaars J.; Strohbach M.W.; Gruber B.; Dormann C.F.	Microsite Conditions Dominate Habitat Selection Of The Red Mason Bee (Osmia Bicornis, Hymenoptera: Megachilidae) In An Urban Environment: A Case Study From Leipzig, Germany	Landscape and Urban Planning	10.1016/j.landurbplan.2011.05.008
2011	Giannini T.C.; Lira-Saade R.; Ayala R.; Saraiva A.M.; Alves-dos-Santos I.	Ecological Niche Similarities Of Peponapis Bees And Non-Domesticated Cucurbita Species	Ecological Modelling	10.1016/j.ecolmodel.2011.03.031
2011	Fadaie SH; Arzani H; Azarnivand H; Nehzati GA; Kaboli SH; Amiri F	A Study Of Range Suitability Model For Apiculture By Using Gis Case Study Taleghan Rangelands	Journal of Applied RS & GIS Techniques in Natural Resource Science	
2010	Gordo O.; Sanz J.J.; Lobo J.M.	Determining The Environmental Factors Underlying The Spatial Variability Of Insect Appearance Phenology For The Honey Bee, Apis Mellifera, And The Small White, Pieris Rapae	Journal of Insect Science	10.1673/031.010.3401
2010	Davis E.S.; Murray T.E.; Fitzpatrick N.; Brown M.J.F.; Paxton R.J.	Landscape Effects On Extremely Fragmented Populations Of A Rare Solitary Bee, Colletes Floralis	Molecular Ecology	10.1111/j.1365-294X.2010.04868.x
2010	Arthur A.D.; Li J.; Henry S.; Cunningham S.A.	Influence Of Woody Vegetation On Pollinator Densities In Oilseed Brassica Fields In An Australian Temperate Landscape	Basic and Applied Ecology	10.1016/j.baae.2010.05.001
2010	Saberioon M.M.; Mardan M.; Nordin L.; Alias M.S.; Gholizadeh A.	Predict Location(S) Of Apis Dorsata Nesting Sites Using Remote Sensing And Geographic Information System In Melaleuca Forest	American Journal of Applied Sciences	10.3844/ajassp.2010.252.259
2010	Estoque R.C.; Murayama Y.	Suitability Analysis For Beekeeping Sites In La Union, Philippines, Using Gis And Multi-Criteria Evaluation Techniques	Research Journal of Applied Sciences	10.3923/rjasci.2010.242.253
2010	Suzuki-Ohno Y.; Inoue M.N.; Ohno K.	Applying Geographic Profiling Used In The Field Of Criminology For Predicting The Nest Locations Of Bumble Bees	Journal of Theoretical Biology	10.1016/j.jtbi.2010.04.010
2009	Patiny S.; Michez D.; Kuhlmann M.; Pauly A.; Barbier Y.	Factors Limiting The Species Richness Of Bees In Saharan Africa	Bulletin of Entomological Research	10.1017/S0007485308006433
2009	Zhu Y.; Zhao J.; Liu S.; Yue E.	Design Of Bee Products Quality Monitoring Information Service Platform	IFIP Advances in Information and Communication Technology	10.1007/978-1-4419-0213-9_66
2009	Sandes Jr. R.L.; Oliveira C.L.; Ferreira E.S.; Cruiff E.; Tavares C.; Santos A.C.B.; Franke C.R.; Bavia M.E.	Spatial Analysis Of Migrating Apis Mellifera Colonies In Salvador, Bahia, Brazil	Geospatial Health	10.4081/gh.2009.215
2008	Serrano S.; Jiménez-Hornero F.J.; Gutiérrez de Ravé E.; Jodral M.L.	Gis Design Application For "Sierra Morena Honey" Designation Of Origin	Computers and Electronics in Agriculture	10.1016/j.compag.2008.07.001
2008	Li S.; Zhu Y.; E Y.; Liu S.	Status Quo Of Quality Safety Of Bee Products And Construction Of Whole-Process Traceability System	Nongye Gongcheng Xuebao/Transactions of the Chinese Society of Agricultural Engineering	
2008	Maris N.M.N.; Mansor S.; Shafri H.Z.M.	Apicultural Site Zonation Using Gis And Multi-Criteria Decision Analysis	Pertanika Journal of Tropical Agricultural Science	
2007	Fitzpatrick U.; Murray T.E.; Paxton R.J.; Breen J.; Cotton D.; Santorum V.; Brown M.J.F.	Rarity And Decline In Bumblebees - A Test Of Causes And Correlates In The Irish Fauna	Biological Conservation	10.1016/j.biocon.2006.11.012
2007	Winfree R.; Griswold T.; Kremen C.	Effect Of Human Disturbance On Bee Communities In A Forested Ecosystem	Conservation Biology	10.1111/j.1523-1739.2006.00574.x
2007	Herrmann F.; Westphal C.; Moritz R.F.A.; Steffan-Dewenter I.	Genetic Diversity And Mass Resources Promote Colony Size And Forager Densities Of A Social Bee (Bombus Pascuorum) In Agricultural Landscapes	Molecular Ecology	10.1111/j.1365-294X.2007.03226.x

2007	Abdelkader B; Khalladi M; Khelloufi B; Tayeb S	Preparation Of Beekeeping Potentiality Map Using Rs And Gis, Mounts Of Beni Chougrane, Mascara, Algeria	ARAB GULF JOURNAL OF SCIENTIFIC RESEARCH	
2007	Cordoni Guido; Spagnuolo Loreta Maria	Development Of An Experimental Geographic Information System For Bee-Keeping In The Marche Region Of Italy	Veterinaria Italiana	
2006	Diekötter T.; Walther-Hellwig K.; Conradi M.; Suter M.; Frankl R.	Effects Of Landscape Elements On The Distribution Of The Rare Bumblebee Species Bombus Muscorum In An Agricultural Landscape	Biodiversity and Conservation	10.1007/s10531-004-2932-9
2006	Janssens X.; Bruneau É.; Lebrun P.	Prediction Of The Potential Honey Production At The Apiary Scale Using A Geographical Information System (Gis); [Prévision Des Potentialités De Production De Miel À L'Échelle D'Un Rucher Au Moyen D'Un Système D'Information Géographique]	Apidologie	10.1051/apido:2006006
2006	Biesmeijer J.C.; Roberts S.P.M.; Reemer M.; Ohlemüller R.; Edwards M.; Peeters T.; Schaffers A.P.; Potts S.G.; Kleukers R.; Thomas C.D.; Settele J.; Kunin W.E.	Parallel Declines In Pollinators And Insect-Pollinated Plants In Britain And The Netherlands	Science	10.1126/science.1127863
2004	Kremen C.; Williams N.M.; Bugg R.L.; Fay J.P.; Thorp R.W.	The Area Requirements Of An Ecosystem Service: Crop Pollination By Native Bee Communities In California	Ecology Letters	10.1111/j.1461-0248.2004.00662.x
2004	Mazzone P.; Caprio E.; Cringoli G.	Varroa Mites In The Apiaries Of Campania Region; [Presenza E Diffusione Di Varroa Negli Alveari Della Campania]	Parassitologia	
2004	Berardinelli I; Della Vedova G	Use Of Gis In The Management Of Apiculture: Preliminary Note [Geographic Information System; Friuli-Venezia Giulia]	APOidea - Rivista Italiana di Apicoltura	
2003	Filis I.V.; Sabrakos M.; Yialouris C.P.; Sideridis A.B.; Mahaman B.	Gedas: An Integrated Geographical Expert Database System	Expert Systems with Applications	10.1016/S0957-4174(02)00080-5
2002	Steffan-Dewenter I.; Münzenberg U.; Bürger C.; Thies C.; Tscharntke T.	Scale-Dependent Effects Of Landscape Context On Three Pollinator Guilds	Ecology	10.1890/0012-9658(2002)083[1421:SDEOLC]2.0.CO;2
2001	Christopher Brown J.; Albrecht C.	The Effect Of Tropical Deforestation On Stingless Bees Of The Genus Melipona (Insecta: Hymenoptera: Apidae: Meliponini) In Central Rondonia, Brazil	Journal of Biogeography	10.1046/j.1365-2699.2001.00583.x
2001	Jo Myung-Hee; Kim Joon-Bum; Baek Seong-Ryul	Selection Technique For Honey Plant Complex Area Using Landsat Image And Gis	22nd Asian Conference on Remote Sensing	
2000	Ward C.A.; Starks S.A.	Approach To Predict Africanized Honey Bee Migration Using Remote Sensing	Computers and Electrical Engineering	10.1016/S0045-7906(99)00028-2
1998	Dippel K; Büchler R; Müller KH; Walther J	Darstellung Eines Landesweiten Bienenkatasters Für Hessen Mit Hilfe Geographischer Informationssystem Gis	Apidologie	
1996	Richardson A.J.; Wiegand C.L.; Anderson G.L.; Gerbermann A.H.; Bray M.; Summy K.R.; Sugden E.A.	Six Exemplary Applications Of Gis Technology To Subtropical Texas Agriculture And Natural Resources	Geocarto International	10.1080/10106049609354523
1992	Makela M.	Africanized Bees On The Move	GIS World	
1992	Winston M.L.	The Biology And Management Of Africanized Honey Bees	Annual Review of Entomology	10.1146/annurev.en.37.010192.001133
1977	Taylor OR Jr	The Past And Possible Future Spread Of Africanized Honeybees In The Americas	Bee World	10.1080/0005772X.1977.11097632
